# Destroying pathogen-tumor symbionts synergizing with catalytic therapy of colorectal cancer by biomimetic protein-supported single-atom nanozyme

**DOI:** 10.1038/s41392-023-01491-8

**Published:** 2023-07-21

**Authors:** Xinyue Wang, Qian Chen, Yefei Zhu, Kairuo Wang, Yongliang Chang, Xiawei Wu, Weichao Bao, Tongcheng Cao, Hangrong Chen, Yang Zhang, Huanlong Qin

**Affiliations:** 1 Nanomedicine and Intestinal Microecology Research Center, Shanghai Tenth People’s Hospital, School of Medicine, Tongji University, Shanghai, 200072 PR China; 2 State Key Laboratory of High Performance Ceramics and Superfine Microstructure, Shanghai Institute of Ceramics, Shanghai, 200050 China; 3 Shanghai Key Lab of Chemical Assessment and Sustainability, School of Chemical Science and Engineering, Tongji University, 1239 Siping Road, Shanghai, 200092 China; 4 Precision Medicine Center, Taizhou Central Hospital, 999 Donghai Road, Taizhou, 318000 Zhejiang China

**Keywords:** Drug development, Gastrointestinal cancer, Cancer microenvironment

## Abstract

The crucial role of intratumoral bacteria in the progression of cancer has been gradually recognized with the development of sequencing technology. Several intratumoral bacteria which have been identified as pathogens of cancer that induce progression, metastasis, and poor outcome of cancer, while tumor vascular networks and immunosuppressive microenvironment provide shelters for pathogens localization. Thus, the mutually-beneficial interplay between pathogens and tumors, named “pathogen-tumor symbionts”, is probably a potential therapeutic site for tumor treatment. Herein, we proposed a destroying pathogen-tumor symbionts strategy that kills intratumoral pathogens, *F. nucleatum*, to break the symbiont and synergize to kill colorectal cancer (CRC) cells. This strategy was achieved by a groundbreaking protein-supported copper single-atom nanozyme (BSA-Cu SAN) which was inspired by the structures of native enzymes that are based on protein, with metal elements as the active center. BSA-Cu SAN can exert catalytic therapy by generating reactive oxygen species (ROS) and depleting GSH. The in vitro and in vivo experiments demonstrate that BSA-Cu SAN passively targets tumor sites and efficiently scavenges *F. nucleatum* in situ to destroy pathogen-tumor symbionts. As a result, ROS resistance of CRC through elevated autophagy mediated by *F. nucleatum* was relieved, contributing to apoptosis of cancer cells induced by intracellular redox imbalance generated by BSA-Cu SAN. Particularly, BSA-Cu SAN experiences renal clearance, avoiding long-term systemic toxicity. This work provides a feasible paradigm for destroying pathogen-tumor symbionts to block intratumoral pathogens interplay with CRC for antitumor therapy and an optimized trail for the SAN catalytic therapy by the clearable protein-supported SAN.

## Introduction

The established connection between cancer and microbes can trace back to four millennia ago.^1^ With the development of sequencing technologies, the importance of microbes in cancer and cancer treatment has received increasing acknowledgements recently, especially in the large intestine.^2^ Colorectal cancer (CRC) has been the third most common cancer worldwide.^3^ The microbiota is recognized as the capital carcinogenesis of CRC and influences therapeutic efficacy.^4^ As ample evidence reveals the existence of intratumoral bacteria and their active performance,^2^ the symbionts of pathogens and CRC, here defined as the pathogen-tumor symbionts, have gained substantial attention, while the exploration of the intricate interplay between pathogens and CRC has been on the march.^5^ Some bacterial strains such as polyketide synthase (pks) *Escherichia. coli*, enterotoxigenic *Bacteroides fragilis* (ETBF), and *Enterococcus faecalis* have been found to induce a chronic inflammatory milieu abundant with nuclear kappa B, interleukin, tumor necrosis factor, and so forth that conduce to cell proliferation and canceration.^6^ The changed intestinal niche then further attracts opportunistic bacteria localization.^7^ As mutual benefits, certain bacterial species can secret specific toxins that suppress apoptosis, stimulate proliferation and drive immune dysregulation to support tumor growth,^8^ while tumor vascular networks and immunosuppressive tumor microenvironment provide shelters for bacteria.^2^ Additionally, increasing evidences reveal that intratumoral bacteria enhance the survival of tumor cells in circulation and promote metastasis.^9^ From this perspective, interfering with intratumoral pathogens, thereby destroying pathogen-tumor symbionts is a potential therapeutic strategy for anticancer.

It is widely acknowledged that *Fusobacterium (F.) nucleatum* is abundant in CRC tissues, which plays a durative role in the pathogenesis,^10^ progression,^11^ metastasis,^12^ and poor outcome.^13^ The mechanisms relate to FadA adhesion and the TLR4/AKT signaling activation that assists cancer development.^14^ Simultaneously, *F. nucleatum* induces autophagy pathway up-regulation by TLR4-MYD88 signaling and genomic loss of miR-18a and miR-4802, which works as a protection mechanism from chemotherapy and oxidative damage.^15–17^ A previous study found that when colon cancer xenograft tumor-bearing mice were treated with the antibiotic metronidazole, *F. nucleatum* load decreased, and the tumor growths were diminished concomitantly.^18^ Therefore, scavenging of intratumoral *F. nucleatum* is beneficial for treating CRC.^19^

In the biological fields, copper indispensably involve in natural biological processes such as protein composition and biocatalysis activation.^20^ Copper-based nanomaterials can escape the ion pumps, causing abundant copper to be endocytosed to cytoplasm.^20^ Moreover, copper-based nanoplatforms can produce ROS with depleting GSH to accumulate oxidative stress for catalytic therapy due to copper’s multivalent nature.^21–23^ Single-atom nanozymes (SANs) have high catalytic activity due to the large specific surface area and extreme atom utilization,^24,25^ which plays versatile functions in sensing, degradation, tumor inhibition, and antibacterial.^26^ For example, a PEGylated manganese-based SAN (Mn/PSAE) was reported to treat breast cancer model.^27^ Fe single atoms-anchored defective carbon dots in PEGylated porous silica nanoreactors (Fe/CDs@PPSNs) could effectively treat hepatocellular carcinoma model.^24^ A hollow N-doped carbon sphere doped with a single-atom copper species (Cu-HNCS) was also designed for anticancer.^28^ SANs respond to the specific tumor microenvironment (TME) for targeted catalytic therapy have shown brilliant prospect in cancer treatment and provides a prerequisite for killing intratumoral pathogens. SANs now available are dominantly based on carbon-based supports such as N-doped carbon, carbon nitride and MOF-carbon. Some other metal single atoms dope on noncarbon-based supports such as transition metal oxides.^29^ Rare single atoms doped on protein have been reported yet. Protein-based materials typically exhibit benefits such as biocompatibility, biodegradability, and an abundance of functional groups, all of which contribute to their successful clinical implementation. Therefore, the design and development of SANs highly simulating natural enzymes structures that are based on protein with metal elements as active centers potentially expand the types of artificial enzymes and provides an example for the synthesis of high-performance protein-based SANs.

**Fig. 1:**
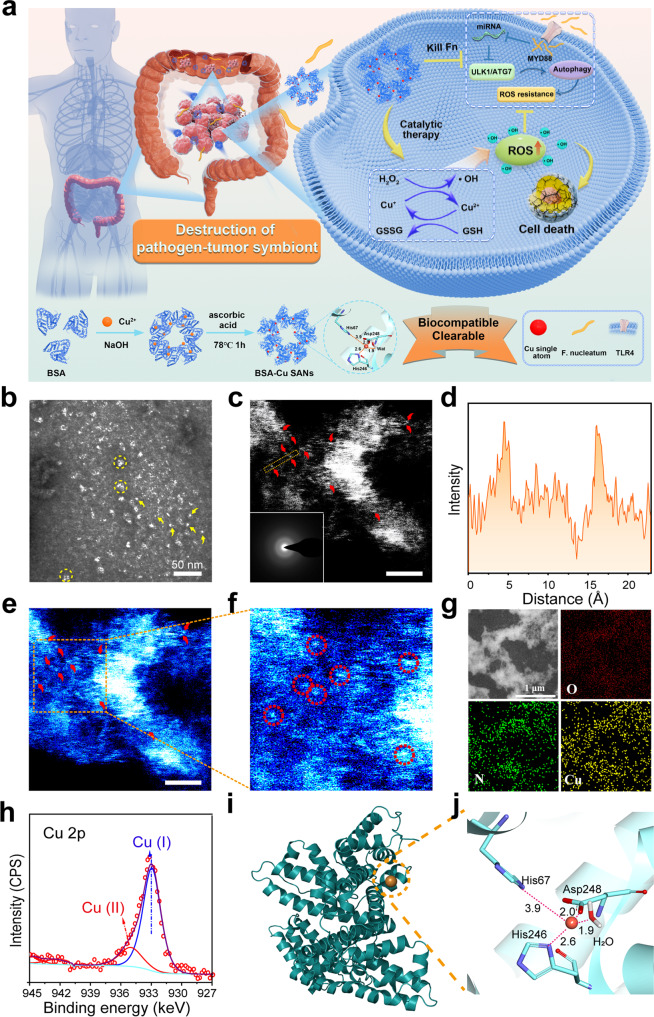
Formation and characterization of BSA-Cu SAN. **a** Schematic illustration of the synthesis of BSA-Cu SAN and its function of destroying pathogen-tumor symbionts for antitumor therapy. **b** Negative-stain electron microscopy image of BSA-Cu SAN. White bots represent BSA-Cu SAN, some of which are marked by yellow arrows. Minority of agglomerates circle in yellow. **c** Atomic HAADF image of BSA-Cu SAN with SAED pattern inset in the bottom left corner and partial single Cu atoms highlighted in yellow dash circles. **d** The intensity spectrum image along the distance in the yellow dash tangle in (**c**). **e** Pseudo-color image of corresponding intensity of (**c**), scale bars = 2 nm. **f** the enlarged image of (**e**). **g** Elemental mapping of BSA-Cu SAN. **h** Cu 2p XPS spectra of BSA-Cu SAN. **i** Binding site of Cu^+^ on BSA based on the highest docking score. **j** Protein-ligand interaction diagram for Cu^+^

Herein, a destroying pathogen-tumor symbionts strategy that directly eliminates intratumoral pathogens, *F. nucleatum*, to break the symbionts and synergize to kill CRC cells was proposed. And this strategy was performed by a groundbreaking protein-supported copper single-atom nanozyme (BSA-Cu SAN) via N and O atoms in BSA coordinated with Cu single atoms (Fig. 1a). The obtained BSA-Cu SAN passively targets tumor sites and efficiently catalyzed H_2_O_2_ into toxic •OH and simultaneously depleted GSH in the TME for redox imbalance. As a notorious intratumoral pathogen, *F. nucleatum* could be validly inhibited, and as a positive feedback, *F. nucleatum*-mediated ROS resistance could be relieved and consequently promoted CRC cell death under a single reagent of BSA-Cu SAN. Noticeably, BSA-Cu SAN with protein base was biocompatible and cleared by renal, successfully solving the concern of accumulated toxicity of N-doped carbon-based SAN. Consequently, this work provides a practical strategy that destroys pathogen-tumor symbionts for antitumor therapy and broadens the sort of SANs by developing protein-supported copper single atoms that make a step forward in the clinical transformation of SANs for catalytic therapy.

## Results

### Synthesis and characterization of BSA-Cu SAN

Inspired by the structures of native enzymes that are based on protein, and metal elements as the active center, BSA-Cu SAN was synthesized under moderate temperatures (Fig. 1a). Ascorbic acid was used as a reducing agent to convert Cu^2+^ into Cu^+^ to construct the copper SAN with high catalytic activity. The copper loading at 5.4 wt% was selected for best dispersion and stability since higher concentrations of copper (10.8, 21.7, and 43.4 wt%) produced precipitates (Supplementary Fig. 1). Negative-stain electron microscopy revealed that BSA-Cu SAN was amorphous with a size of 7.6 ± 0.9 nm (Fig. 1b). The amorphous protein matrix was also confirmed by the diffusion diffraction ring in the inset select area electron diffraction (SAED) pattern (Fig. 1c). Atomic high-angle annular dark-field (HAADF) images, produced by aberration-corrected transmission electron microscopy (AC-TEM), displayed that isolated copper atoms (marked in red arrows) were uniformly dispersed in the protein matrix (Fig. 1c). The two high-intensity peaks in the spectrum (the yellow rectangle box) in Fig. 1c corresponded to the two bright spots due to their large atomic number (Fig. 1d). There are well-marked bright dots in the corresponding intensity image and its enlarged area that show the atomic dispersion of Cu single atoms (Fig. 1e, f). The average diameter of copper dots was measured at ~1.2 Å, which was dissimilar to the distance of the Cu–Cu covalent bond at 2.7 Å.^30^ This observation supported the single-atom coordination of copper. No copper particle with crystalline structure was detected in BSA-Cu SAN at 5.4 wt% concentrations of copper, while higher loading of copper inclined to a larger size and cluster trend (Supplementary Figs. 2 and 3). This observation confirmed the form of single copper atoms, which was validated by energy-dispersive X-ray spectroscopy (EDS) (Supplementary Fig. 4). Element mappings revealed N and O in the protein matrix and Cu were homogeneously distributed (Fig. 1g). Based on Fourier transform infrared (FT-IR) spectra (Supplementary Fig. 5), the bands at 1656 and 1538 cm^−1^ confirmed the existence of BSA.^31^ The Raman spectra of BSA-Cu SAN (Supplementary Fig. 6) also verified the presence of the amide bond. Meanwhile, the average zeta potential of −40 eV symbolized the ideal stability (Supplementary Fig. 7). Since the nanoplatform stability in an aqueous solution is crucial for potential in vivo application, the stability of BSA-Cu SAN was investigated. The results revealed that BSA-Cu SAN retained its original character without precipitation and provided uniform UV-vis spectra for at least 4 weeks (Supplementary Fig. 8). Aside from water, PBS, FBS, DMEM and complete medium which contain 10% FBS and penicillin (100 units/ml) and streptomycin (100 μg/ml) were utilized to simulate body liquid. There was typical Tyndall effect in these five kinds of liquid (Supplementary Fig. 9). Dynamic light scattering (DLS) analysis demonstrated that the size of BSA-Cu SAN was about 15 nm in water (Supplementary Fig. 10), which was compatible with the result of negative-stain electron microscopy (Fig. 1b) considering the good hydrophile of BSA. DLS of BSA-Cu SAN in different solution remained stable for at least 7 days, suggesting significant stability and favorable dispersity of BSA-Cu SAN. X-ray photoelectron spectroscopy (XPS) spectra (Fig. 1h) showed the peak of Cu 2p core energy was at 932.9 eV, indicating the form of monovalent copper, referring 934.7 and 932.3 eV for Cu^2+^ and Cu^+^, respectively.

The binding of Cu^+^ to BSA was studied by molecular docking simulations. There were ten potential binding sites, in which the model of Cu^+^ coordinating with two nitrogen atoms of histidine imidazole rings (His246 and His67) to form Cu-N bonds as well as with the carboxylate oxygen atom of Asp248 and H_2_O to form Cu-O bonds got the highest docking score of −5.286 kcal/mol (Fig.1i, j, Supplementary Fig. 11 and Supplementary Table 1). Briefly, Cu^+^ coordinates with two N atoms of histidine imidazole rings and two oxygen atoms of aspartic acid and H_2_O. Such Cu-N and Cu-O bonds in synthetic BSA-Cu SAN are similar to many native copper-containing enzymes, such as galactose oxidase and ascorbate oxidase.

**Fig. 2:**
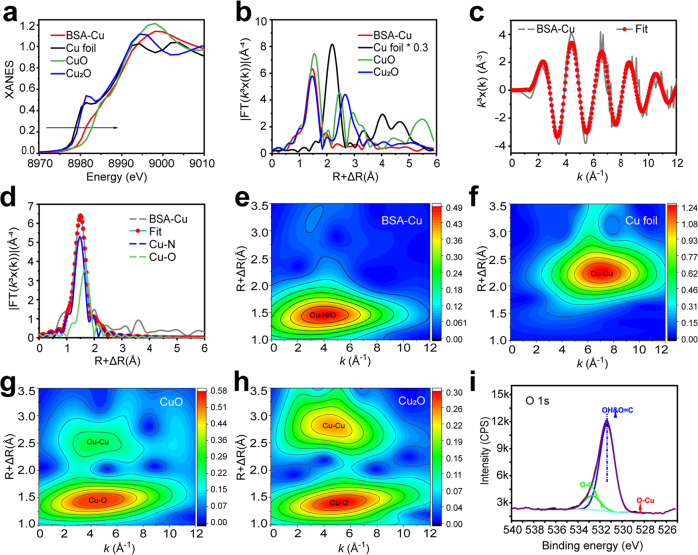
Atomic structural characterization of BSA-Cu SAN. **a** Cu K-edge XANES spectra and **d** Fourier-transform EXAFS spectra of BSA-Cu SAN, Cu foil, CuO, and Cu_2_O. **c**, **d** The corresponding EXAFS fitting of BSA-Cu SAN at **c** K space and **d** R space. **e**–**h** Wavelet transformation of Cu K-edge EXAFS of **e** BSA-Cu SAN, **f** Cu foil, **g** CuO, and **h** Cu_2_O. **i** O 1s XPS spectra of BSA-Cu SAN

Synchrotron radiation was conducted to ascertain the fine structure of the isolated Cu atoms in BSA-Cu SAN. The X-ray absorption near-edge structure (XANES) spectra were applied to identify the oxidation state. The BSA-Cu SAN profile was located between Cu_2_O and CuO in the Cu K-edges XANES (Fig. 2a), indicating that the valence state of Cu was between +1 and +2, which coincidented with the result of XPS. The Fourier transformed extended X-ray absorption fine structure (FT-EXAFS) spectra were employed to realize the adjacent atoms and coordination structure of Cu sites. The FT-EXAFS spectrum demonstrated that the Cu K-edge profile of BSA-Cu SAN prominently peaked at ~1.5 Å (Fig. 2b), attributing to Cu-N coordination,^32^ which was quite different from that of Cu–Cu (~2.2 Å) in metallic copper and oxidized copper.^33^ According to the fitted data (Fig. 2c, d and Supplementary Table 2), the first shell coordination number for Cu-N/O was 4.2 ± 0.5, reflecting four coordinate bonds. The numbers of the first shell coordination for Cu-N and Cu-O were 2.2 ± 0.2 and 1.7 ± 0.4, respectively, deducing the structure of CuN_2_O_2_. Additionally, the higher resolution Morlet wavelet transformation (WT, *κ* = 10, and *σ* = 1) of Cu K-edge oscillations exhibited that the contour plot of BSA-Cu SAN occupied one maximum k-space at ~3.9 Å^−1^ (Fig. 2e). It was well aligned with Cu-N/O and distinguished from Cu-foil (Fig. 2f) and Cu_x_O (Fig. 2g, h). No lattice O^2−^ species were displayed in the O 1s spectrum (Fig. 2i), and no clusters were observed in TEM, indicating the absence of Cu_x_O clusters. Therefore, this study validated that Cu was atomically coordinated with N and O atoms in BSA-Cu SAN.

### Enzyme-like activity and GSH depletion of BSA-Cu SAN

**Fig. 3:**
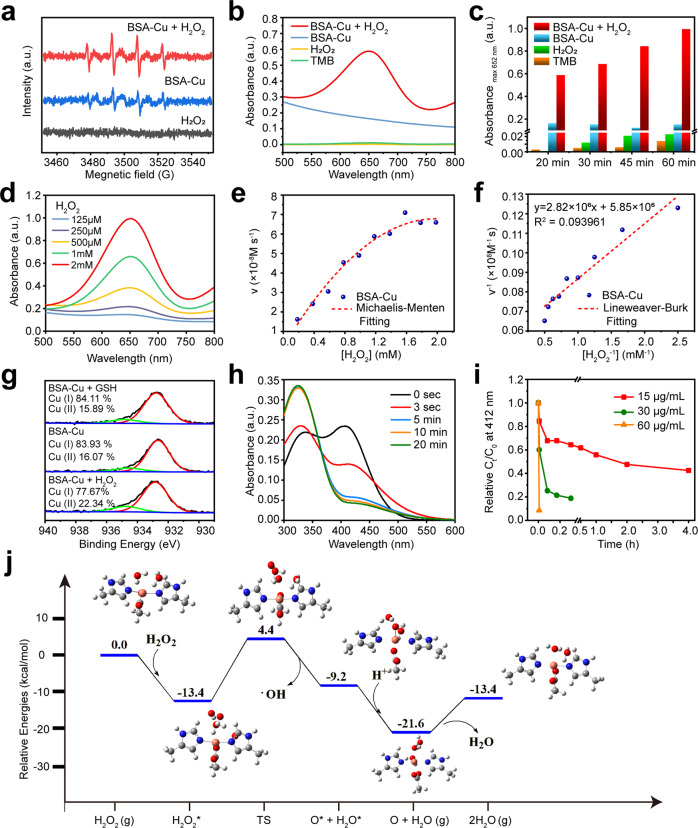
Enzyme-like activity and GSH depletion performance of BSA-Cu SAN. **a** ESR spectra of H_2_O_2_, BSA-Cu SAN, and BSA-Cu SAN + H_2_O_2_. **b** Absorption of TMB oxidized by H_2_O_2_ and BSA-Cu SAN with or without H_2_O_2_. **c** UV-vis absorbance at 652 nm of TMB reacted with different samples for 20, 30, 45, and 60 min. **d** TMB absorption in the presence of 30 ug/mL Cu of BSA-Cu SAN and different concentrations of H_2_O_2_. **e** Michaelis-Menten kinetics and **f** Lineweaver-Burk plot of BSA-Cu SAN. **g** High-resolution Cu 2p XPS spectra of BSA-Cu SAN treated with H_2_O_2_ or GSH for 1 h. **h**, **i** GSH depleting ability of BSA-Cu SAN at **h** varied times and **i** concentrations. **j** DFT studies on the peroxidase-like performance of BSA-Cu SAN. The detailed data is presented in Supplementary Table 4

To validate the catalytic performance of BSA-Cu SAN, the electron spin resonance (ESR) was used to detect •OH by 5,5-dimethyl-1-pyrroline N-oxide (DMPO) as the spin trap. As expected, the ESR spectra showed a typical 1:2:2:1 multiple peak of •OH, indicating that BSA-Cu SAN could catalyze H_2_O_2_ to •OH owing to a Fenton-like reaction (Fig. 3a). Then, 3,3′,5,5′-tetramethylbenzidine (TMB) was used to further explore the catalytic activity of BSA-Cu SAN on the strength of the chromogenic reaction. The •OH oxidated TMB from a colorless transparent liquid to blue color with a characteristic absorbance peak at 652 nm. Compared with the BSA-Cu SAN group, the BSA-Cu SAN with H_2_O_2_ group had a stronger absorption peak at 652 nm, suggesting that BSA-Cu SAN could efficiently produce •OH by the Fenton-like reaction (Fig. 3b). As shown in Fig. 3c, BSA-Cu SAN catalyzed •OH generation in a time-dependent manner, and the •OH yield increased gradually within 1 h, demonstrating that BSA-Cu SAN had high catalytic capacity for at least 1 h. Considering the concentrations of catalysts and substrates would affect the reaction rate, the ROS production at various concentrations of BSA-Cu SAN or H_2_O_2_ was measured. With the increase of BSA-Cu SAN or H_2_O_2_ concentrations, the production of •OH obviously increased at equivalent exposure time (Fig. 3d and Supplementary Fig. 12), indicating the catalyst (BSA-Cu SAN) and substrate (H_2_O_2_) concentrations affected ROS production. Given the weak acid of the TME, the catalytic activity of BSA-Cu SAN under various pH conditions was assessed. The weak acid condition was more suitable for BSA-Cu SAN catalysis (Supplementary Fig. 13), making BSA-Cu SAN respond to the weak acid environment of the tumor for specific nanocatalytic therapy and reduce the damage to normal tissues. Then, the Michaelis–Menten kinetics investigated the detailed catalytic performance of BSA-Cu SAN in pH 6.5. After recording the relative absorbance data and calculation, the Michaelis–Menten curves and Lineweaver-Burk plot, which was the linear double-reciprocal plot of the Michaelis–Menten equation, were produced (Fig. 3e, f). BSA-CuS acts as a contrast due to the similar components. The maximum velocity (*V*_max_) was calculated to be 17.09 × 10^−8^ M s^−1^ for BSA-Cu SAN, *K*_m_ = 0.48 mM and *k*_cat_ = 3.65 × 10^−3^ s^−1^, which significantly exceeded that of BSA-CuS, whose *V*_max_ = 3.52 × 10^−8^ M s^−1^, *K*_m_ = 1.33 mM and *k*_cat_ = 7.10 × 10^−5^ s^−1^ (Supplementary Fig. 14). This result reflected the superiority of copper single atoms to copper ions (Supplementary Table 3). Besides, GSH, a reducing agent and free radical scavenger which is highly expressed in the tumor tissues, is detrimental to nanocatalytic therapy by depleting ROS, hence the effect of GSH on the catalytic reaction of BSA-Cu SAN was investigated. Due to GSH reacting with both Cu (II) and H_2_O_2_, the high concentrations of GSH inhibited TMB oxidation, indicating that GSH depletion was beneficial for nanocatalytic therapy (Supplementary Fig. 15). The reaction preferentially reduced Cu (II) rather than H_2_O_2,_^28^ and GSH promoted the catalytic reaction of BSA-Cu SAN at relatively low GSH concentrations due to Cu (II) reduction by GSH to Cu (I) with higher catalytic activity.^34^ XPS spectra of BSA-Cu SAN were measured before and after the reaction with H_2_O_2_ and GSH to prove the change in copper’s valence state in BSA-Cu SAN. There was a deviation in the Cu 2p XPS spectra from 932.36 to 932.48 eV when BSA-Cu SAN incubated with H_2_O_2_ for 1 h, and the ratio of Cu (I) changed from 83.93 to 77.67%. After the reaction with GSH, Cu 2p XPS spectra of BSA-Cu SAN deviated to 932.22 eV, and Cu (I) increased to 84.11% (Fig. 3g), validating the valency transformation of Cu. Then, the GSH depletion was also explored by 5,5′-dithiobis-(2-nitro-benzoic acid) (DTNB). There was a sharp drop at 412 nm, which was the characteristic peak of DTNB, when BSA-Cu SAN (30 µg/mL Cu) was added to 1 mM GSH solution, and the characteristic peak at 412 nm was negligible after 5 min. These observations implied that GSH was drained by BSA-Cu SAN (Fig. 3h). Even lower concentrations of BSA-Cu SAN (15 μg/mL Cu) effectively depleted GSH over time (Fig. 3i). Therefore, BSA-Cu SAN competently generated ROS via catalytic reaction and consumed GSH. It also contributed to ROS accumulation and persisted high catalytic activity of low-valent copper.

The fine coordination structure helps to understand the reaction mechanism. The specific catalytic mechanism of BSA-Cu SAN was analyzed by density functional theory (DFT) calculations. As shown in Fig. 3j and Supplementary Fig. 16, H_2_O_2_ is absorbed on the copper active site with the absorption energy of −13.4 kcal/mol. Then the absorbed and activated H_2_O_2_ is dissociated to form a transition state of OH-OH species with the energy barriers of 17.8 kcal/mol. Next, a free hydroxyl radical is released, and the residual OH absorbs H^+^ to form H_2_O. These results indicate that BSA-Cu SAN can act as a peroxidase-like nanozyme at room temperature.

### Intervening *Fusobacterium nucleatum* effect of BSA-Cu SAN

**Fig. 4:**
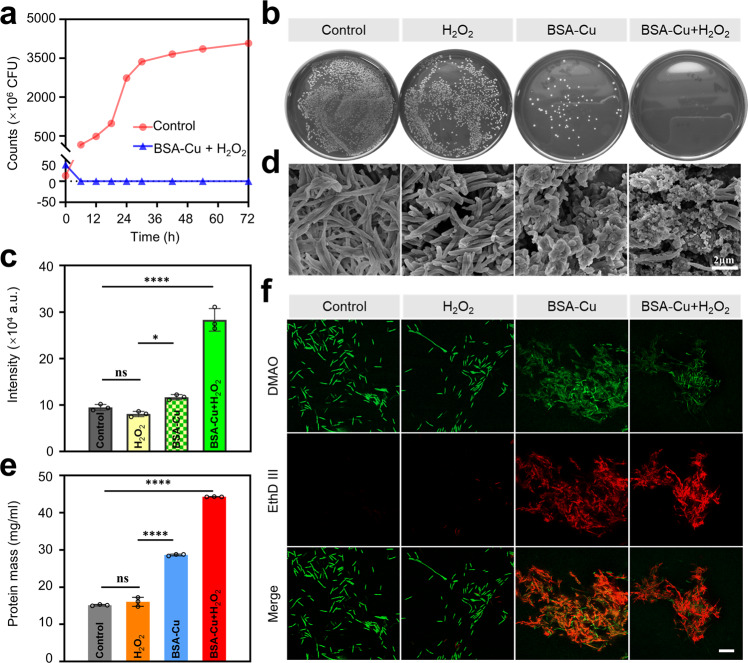
In vitro antibacterial performance of BSA-Cu SAN on *F. nucleatum*. **a** Grow curves of F. nucleatum in normal culture media or culture media containing 45 μg/mL Cu of BSA-Cu SAN and 100 μM H_2_O_2_. **b** Representative photographs of *F. nucleatum* treated with different groups for 6 h (100 μM H_2_O_2_, 45 μg/mL Cu of BSA-Cu SAN, and BSA-Cu SAN + H_2_O_2_), and then inoculated on solid Columbia Blood Agar Plates at 37 ℃ in the anaerobic chamber for 3 days. **c** Fluorescence intensity of *F.nucleatum* stained with DCFH-DA probe after the same treatment as (**b**) for 6 h, then detected by a microplate reader. **d** SEM images of *F. nucleatum* treated with H_2_O_2_, BSA-Cu SAN, and BSA-Cu SAN + H_2_O_2_. **e** The protein leakage from *F. nucleatum* after different treatments for 6 h. **f** CLSM images of live/dead bacteria stained with DMAO/EthD III after the same treatment as before. Bar =10 μM. Data are presented as mean ± SD. Statistical analysis was performed via one-way ANOVA. *p < 0.05, **p < 0.01, ***p < 0.001, ****p < 0.0001

Due to the negative role of *F. nucleatum* in CRC, destroying pathogen-tumor symbionts is a practical approach to block the interplay between pathogens and tumors. Therefore, the antibacterial function of BSA-Cu SAN was investigated. The growth curve drawn by the plate-counting method confirmed that BSA-Cu SAN + H_2_O_2_ completely inhibited *F. nucleatum* proliferation (Fig. 4a). For a more detailed and intuitive evaluation, *F. nucleatum* in the logarithmic growth phase was incubated with different treatments for 4 h. Then, these were collected to spread on the Columbia blood agar plates. Dense *F. nucleatum* colonies in the control group, but a significant decrease in the count of colonies in the BSA-Cu SAN group were observed (Fig. 4b). No colonies appeared when *F. nucleatum* was co-incubated with BSA-Cu SAN + H_2_O_2_, which was attributed to the catalytic property of BSA-Cu SAN. The 2,7-dichlorodihydrofluorescein diacetate (DCFH-DA) probe detected ROS generation. The fluorescent intensity of DCFH verified that the bacteria elimination was positively related to the ROS amount (Fig. 4c). SEM images depicted the morphology of *F. nucleatum* (Fig. 4d). Compared with the control and H_2_O_2_ groups, the morphology of *F. nucleatum* was significantly destroyed with membrane collapsed and broken when *F. nucleatum* encountered BSA-Cu SAN with or without H_2_O_2_. Due to the loss of cell membrane integrity following with the leakage of protein in the intracellular matrix, the protein leakage from *F. nucleatum* after different treatments was detected. *F. nucleatum* treated with BSA-Cu SAN + H_2_O_2_ showed the maximum protein content and that was considerably higher than that of the control and H_2_O_2_ groups (Fig. 4e). Live/dead staining to bacteria displayed similar results that *F. nucleatum* destructively died when confronted with BSA-Cu SAN + H_2_O_2_, which manifested the red staining in the confocal laser scanning microscope (CLSM) images (Fig. 4f). These results proved that BSA-Cu SAN could effectively kill *F. nucleatum*, providing a prerequisite and guarantee for intervention in intratumoral bacterial and destruction of pathogen-tumor symbionts.

### Inhibition of pathogens-promoted colorectal cancer cells by BSA-Cu SAN

**Fig. 5:**
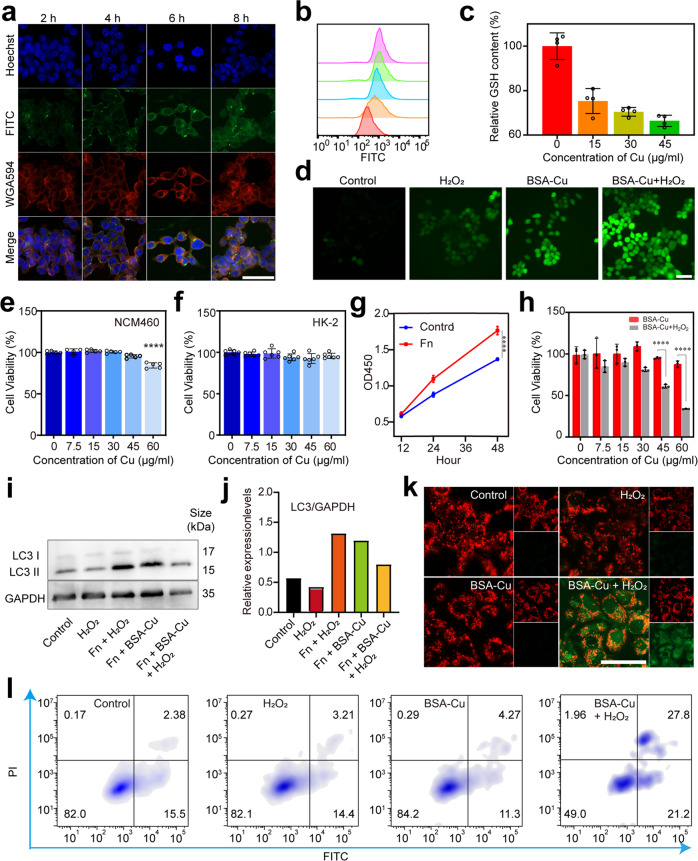
In vitro intervening *F. nucleatum* -induced tumor proliferation with BSA-Cu SAN on HCT116 cells. **a** CLSM images of HCT116 cells incubated with BSA-Cu-FITC containing 45 μg/mL Cu at different times. **b** Uptake of BSA-Cu SAN at different culture times analyzed by flow cytometry. (**c**) Relative contents of GSH in HCT116 cells treated with different concentrations of BSA-Cu SAN for 4 h. **d** Fluorescence images of HCT116 cells stained with DCFH-DA after various treatments with H_2_O_2_ only, BSA-Cu SAN only, and BSA-Cu + H_2_O_2_ for ROS detection. **e** Cell viability of NCM460 cells and **f** HK-2 cells incubated with different concentrations of BSA-Cu SAN for 24 h at pH 7.4. **g** Cell proliferation assay using CCK8. HCT116 cells were cultured with or without *F. nucleatum* (Fn) at different times. **h** Cell viability of HCT116 cells incubated with different concentrations of BSA-Cu SAN with or without 100 μM H_2_O_2_ at pH 6.5 for 24 h. **i** Western blot results of LC3 expression in HCT116 cells after various treatments (control, H_2_O_2_, Fn + H_2_O_2_, Fn + BSA-Cu, Fn + BSA-Cu + H_2_O_2_). **j** Relative expression of LC3 II in (**i**). **k** CLSM images of HCT116 cells stained with JC-1 after various treatments to detect the changes in mitochondrial membrane potential. Red fluorescence represents aggregates, while green fluorescence represents monomers. **l** Cell apoptosis results via flow cytometry analyses of HCT116 cells treated with BSA-Cu SAN with or without H_2_O_2_ . Scale bars = 50 μm. *p < 0.05, **p < 0.01, ***p < 0.001, ****p < 0.0001

To investigate the endocytosis of BSA-Cu SAN, FITC-labeled BSA-Cu SAN was incubated with HCT116 cells and was observed by CLSM. The green fluorescence obviously increased over time (Fig. 5a), representing the uptake of BSA-Cu SAN by cancer cells. Most BSA-Cu SAN was endocytosed in the cytoplasm after incubation for 6 h. The results of flow cytometry analysis were consistent with that of CLSM images, with the highest mean fluorescent intensity (MFI) at 6 h (Fig. 5b and Supplementary Fig. 17), which was the basis for the co-incubation time in the subsequent experiments. HCT116 cells were intervened differently for 6 h, the intracellular GSH level decreased with the increase of BSA-Cu SAN (Fig. 5c). The DCFH-DA probe can detect the •OH generation to validate ROS generation. The group of control and single H_2_O_2_ presented slight fluorescence, while BSA-Cu SAN combined with H_2_O_2_ was the brightest, indicating an abundant •OH production (Fig. 5d). Therefore, BSA-Cu SAN exhibited ideal catalytic behavior to produce ROS and consume GSH at the cellular level.

Next, the toxicity of BSA-Cu SAN to normal cells was investigated by Cell counting kit-8 (CCK8) assay. The viability exhibited no significant decrease after co-incubation with BSA-Cu SAN for 24 h in a normal human colon mucosal epithelial cell line (NCM460) and human renal proximal tubule cells (HK-2) (Fig. 5e, f). This observation proved the low toxicity of BSA-Cu SAN to normal cells within the evaluation concentrations. Since *F. nucleatum* was recognized as a promotor for CRC,^12^ the effect of *F. nucleatum* on HCT116 cell proliferation was investigated by the CCK8 assay (Fig. 5g). Consistent with previous research, HCT116 cells accelerated proliferation when co-incubated with *F. nucleatum* through TLR4/AKT signaling activation according to our previous study.^14^ Therefore, it is reasonable to arrest this mechanism by destroying the pathogen-tumor symbionts strategy. To evaluate the inhibition of pathogen-induced tumor proliferation by BSA-Cu SAN, HCT116 cells were co-cultured with *F. nucleatum* to establish a pathogen-tumor model. Compared with the BSA-Cu SAN and H_2_O_2_ groups, the cell viability significantly decreased with increasing BSA-Cu SAN concentrations combined with H_2_O_2_ at pH 6.5. Furthermore, the cell viability of BSA-Cu SAN (60 μg/mL Cu) + H_2_O_2_ group decreased to only 33.8% (Fig. 5h), indicating that BSA-Cu SAN possessed high therapeutic efficacy, while single H_2_O_2_ was powerless (Supplementary Fig. 18). The images of live/dead cell staining assay provided an intuitive visualization (Supplementary Fig. 19), in which Calcein-AM-stained green fluorescence and PI-stained red fluorescence represented live and dead cells, respectively. Consistent with the CCK8 results, more dead HCT116 cells staining in red with morphological changes were observed in the group of BSA-Cu SAN + H_2_O_2_. Since *F. nucleatum* could up-regulate autophagy which induces ROS resistance and mitigate oxidative damage,^15^ the autophagy levels of HCT116 cells were explored by western blot (WB) after different treatments. LC3 alteration was generally recognized as the gold standard of autophagy.^35^ The WB results revealed that the relative expressive of LC3 II was up-regulated after incubation with *F. nucleatum*, indicating that *F. nucleatum* up-regulated the autophagy level of HCT116 cells. Nevertheless, BSA-Cu SAN + H_2_O_2_ could feasibly relieve the elevated autophagy level triggered by *F. nucleatum* to a relatively normal degree (Fig. 5i, j), which is possibly attributed to the synergistic anti-*F. nucleatum* effect of BSA-Cu SAN and nanocatalysis.

It was reported that multiple copper-containing reagents induced ROS soaring and apoptosis.^36–40^ The turbulence of mitochondrial membrane potential (Δψm) is a sensitive indicator of cell apoptosis, whereby the sustained drop in Δψm is the earliest event during the apoptotic cascade.^41^ The 5,5′,6,6′-tetrachloro-1,1′,3,3′-tetraethylbenzimidazolyl carbocyanine-iodide (JC-1) fluorescent probe was used to detect the Δψm variation. The cells displayed weak red aggregate fluorescence and strong green monomer fluorescence in the presence of BSA-Cu SAN and H_2_O_2_ (Fig. 5k), illustrating that ROS resulted in decreased Δψm. Furthermore, flow cytometry analyzed the apoptosis of HCT116 cells (Fig. 5l). There were slight changes between the BSA-Cu SAN (45 μg/mL Cu) and control groups. However, the BSA-Cu SAN group in the presence of H_2_O_2_ increased the apoptotic cell proportion to more than 63.7% after incubation for 24 h. Therefore, BSA-Cu SAN can suppress pathogen-induced cancer cells by modulating autophagy and inducing apoptosis.

### In vivo antitumor performance

**Fig. 6:**
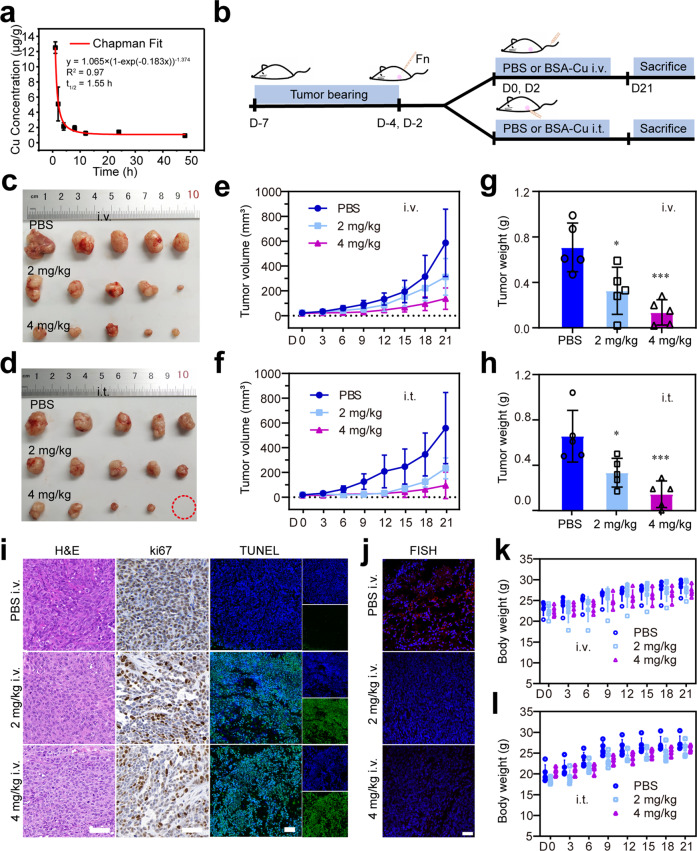
In vivo destroying pathogen-tumor symbionts and antitumor effects of BSA-Cu SAN. **a** The concentrations of Cu in the blood after intravenously injected with BSA-Cu SAN at different time points. *n* = 3, data represent mean ± SD. **b** The schedule of F. nucleatum-tumor models and therapeutic procedures. **c**, **d** The photo of the tumor excised from **c** intravenously injected and **d** intratumorally injected mice after 21 days of treatment. **e**, **f** Tumor volume changes of **e** i.v. and **f** i.t. groups (*n* = 5). **g**, **h** Average tumor weight on the 21st day of treatment. **i** H&E, ki67, and TUNEL immunostaining of representative tumor tissue sections intravenously injected with PBS or different concentrations of BSA-Cu SAN. **j** Images of tumor tissue section in different groups in which the nucleic acid stained blue with DAPI and red for the F. nucleatum probe. **k**, **l** Body weight during therapy. Scale bars = 50 μm

Encouraged by the reliable antibacterial effect and cytotoxicity to pathogens-induced CRC cells of BSA-Cu SAN in vitro, the biodistribution and clearance of BSA-Cu SAN were investigated by fluorescence imaging at first. Cy5.5-labeled BSA-Cu SAN was injected into the HCT116 tumor-bearing mice via the tail vein. The fluorescence signal initially appeared in the liver and kidney at 0.5 h after injection and peaked at 8 h (Supplementary Fig. 20). The fluorescence signal in the kidney was still strong at 24 h and disappeared at 48 h, suggesting that BSA-Cu SAN could be clearable from the body by renal clearance. The fluorescence intensity in the blood exhibited similar results (Supplementary Fig. 21). The half-life time of BSA-Cu SAN was appraised to be 1.55 h by detecting the exact concentrations of Cu at different time points (Fig. 6a). The fluorescence of the main organs and blood at 48 h was equivalent to the initial, indicating the complete clearance of BSA-Cu SAN. It also suggested that it could relieve the concern of heavy metal accumulation in the organs. In the tumor sites, the strong fluorescence signal appeared at 2 h and lasted until 8 h. Therefore, BSA-Cu SAN was accumulated to the tumor relying on the enhanced permeability and retention (EPR) effect, which guarantees subsequent treatment. Subsequently, BALB/c mice of both genders were intravenously injected with PBS or different doses of BSA-Cu SAN (2 or 4 mg/kg Cu) twice in 48 h intervals to investigate the safety of BSA-Cu SAN in vivo. During the observation period of 1 month, the body weights demonstrated a steady growth (Supplementary Fig. 22), and no fatalities occurred before euthanasia. The blood was collected at the endpoint for routine blood tests and serum biochemistry analysis (Supplementary Figs. 23 and 24). All common indexes in routine blood tests and key markers, such as LDH, BUN, UREA, AST, and ALT, were in the normal ranges, and there was no significant difference between the BSA-Cu SAN treated groups and the control groups. No pathological changes were observed in the H&E staining of the main organ sections (Supplementary Fig. 25). These results suggest the reliable biocompatibility of BSA-Cu SAN, potentially providing access to transformation for therapeutic drugs.

The therapeutic effect was further assessed in vivo. A subcutaneous CRC model was constructed using HCT116 cells on BALB/c nude mice (Fig. 6b). *F. nucleatum* was intratumorally injected twice for stable colonization before treatment.^42^ Two days after the second dose of *F. nucleatum*, mice were randomly divided into two cohorts: intratumoral injection (i.t. cohort) and intravenous injection (i.v. cohort). Each cohort involved three groups (*n* = 5). Two dosages of PBS or 2 or 4 mg/kg BSA-Cu SAN were injected intratumorally or intravenously for 48 h. The tumors were harvested for final assessments 21 days after treatment. The photos of tumors gave a visual sense of the tumoral suppression effect of BSA-Cu SAN in both i.t. and i.v. cohorts (Fig. 6c, d). Tracing the tumor sizes depicted that BSA-Cu SAN treatment sustainably inhibited tumor growth (Fig. 6e, f). Furthermore, the tumor growth inhibition value (TGI) of different injected doses (2 or 4 mg/kg) was 58.23 and 82.97% in the i.t. cohort and 46.79 and 76.63% in the i.v. cohort. The tumor weights matched with the tumor volumes, proving satisfied tumor suppression effect of BSA-Cu SAN (Fig. 6g, h). H&E, immunohistochemistry, and immunofluorescence staining were analyzed on these tumor tissues (Fig. 6i and Supplementary Fig. 26). BSA-Cu SAN induced well differentiation, less ki67 positive signal, and significantly stronger TUNEL signal than the control groups, indicating that BSA-Cu SAN depicted the prohibition of proliferation and the impelling of apoptosis.^43^ The fluorescence in situ hybridization (FISH) was carried out to verify the BSA-Cu SAN capacity against *F. nucleatum* in the tumors, in which the red fluorescent bright spot represented *F. nucleatum*. Compared with the control group, the red fluorescent bright spot scarcely appeared in BSA-Cu SAN treated groups, proving that BSA-Cu SAN could effectively scavenge *F. nucleatum* in tumors (Fig. 6j and Supplementary Figs. 27 and 28). Concurrently, the body weight of mice treated with BSA-Cu SAN increased steadily regardless of intratumoral injection or intravenous injection, indicating its low system toxicity (Fig. 6k, l). These results demonstrate that BSA-Cu SAN effectively suppressed tumor growth by the destroying pathogen-tumor symbionts strategy that blocks intratumoral pathogens interplay with CRC through effective catalytic therapy, and it can experience renal clearance, exhibiting potential clinical applications.

## Discussion

The intimate relationship between bacteria and cancer has been gradually recognized in depth. In recent years, scientists have found that bacteria not only exist in virtually all tumor sites but also in immune cells, and the microbiome composition varied significantly in distinct cancer types.^44^ Multiple studies show tumor cells get large profits from specific bacteria, including escape from immunological recognition and attack, promoting proliferation, inducing distant metastasis, and diminishing therapeutic efficacy. The integral view of pathogen-tumor symbionts probably features prominently in the advancement of cancer treatment.

CRC is the most typical representative of pathogen-tumor symbionts, while *F. nucleatum* is one of the most widely accepted intratumoral pathogens in CRC. We construct a pathogen-tumor symbionts model of CRC and use a protein-supported copper single-atom nanozyme to verify the feasibility of destroying pathogen-tumor symbionts strategy. BSA-Cu SAN performs satisfied therapeutic effect by catalyzing H_2_O_2_ to produce ROS, which kill intratumoral *F. nucleatum*, following with reduced autophagy level of CRC cells and enhanced oxidative damage of ROS for apoptosis.

Additionally, BSA endows the SAN with high hydrophia and biocompability. Although BSA-Cu SAN could produce little ·OH detected by ESR, BSA-Cu SAN at the concentrations of 0–60 μg/ml do not show significantly toxic to non-tumor cells (NCM 460 and HK-2 cells). The detection of ·OH does not mean it is toxic to cells absolutely because cells can process a certain level of oxidative stress by reductase or autophagy.^45,46^ In this work, we conducted CCK-8 assay to investigate the toxicity of BSA-Cu SAN to both normal cells and HCT116 cells. The results show that under the experiment concentrations, BSA-Cu SAN is relatively low toxic to cells, as displayed in Fig. 5e, f, h. Only in the present of H_2_O_2_ will BSA-Cu SAN inhibits cell viability owing to the Fenton-like reaction for catalytic therapy. It’s commonly recognized that H_2_O_2_ is highly expressed in TME and low in normal cells. The acid environment in TME also contribute to the catalytic therapy. Meanwhile, relatively small size and high hydrophilia of BSA-Cu SAN make it easy to excrete from the body, which is beneficial for clinical translation.

Based on the mutually-beneficial interplay between pathogens and tumors, scavenging intratumoral pathogens is advantage to cancer treatment. Copper is reported to be broad-spectrum antibacterial metal. Therefore, we deduce that BSA-Cu SAN could also be practical to other kinds of cancers. Directed by the destroying pathogen-tumor symbionts strategy, more therapeutic methods and agents will be developed. Compared with previously reported SAN, BSA-Cu SAN is based on protein which exhibits multiple benefits such as biocompatibility, biodegradability, and an abundance of functional groups. All of these contribute to their successful clinical implementation. Then we use molecular docking simulations method combined with synchrotron radiation to understand the fine structure of BSA-Cu SAN. HAADF images of BSA-Cu SAN showed that Cu element is atomic dispersion and the average diameter of copper dots was measured at ~1.2 Å, which was dissimilar to the distance of the Cu–Cu covalent bond at 2.7 Å. Then, the results of molecular docking simulations indicated that the structure of Cu binding N on His246 and His67, and binding O on Asp248 and H_2_O to form CuN_2_O_2_ got the highest score, indicating Cu^+^ coordinates with two N atoms of histidine imidazole rings and two carboxylate oxygen atoms of aspartic acid and glutamate. Further, according to the results of EXAF spectra fitting data, the first shell coordination number for Cu-N/O was 4.2 ± 0.5, and the numbers of the first shell coordination for Cu-N and Cu-O were 2.2 ± 0.2 and 1.7 ± 0.4, respectively, deducing the structure of CuN_2_O_2_. The wavelet transform analysis also showed the absence of Cu–Cu for Cu foil and for copper oxide. As previously reported, single-atom nanozymes is the nanomaterials which possess intrinsic enzyme-like activities with exclusively single metal atoms as active centers on support, without homoatomic metal-metal bond.^47^ Based on the above results and discussion, we speculate BSA-Cu as copper single-atom nanozymes.

In conclusion, a destroying pathogen-tumor symbionts strategy is proposed in the hope of blocking pathogen-tumor interplay which is a troubling promotor for tumor progression. Catalytic therapy is adopted for response in the TME by novel protein-supported copper single-atom nanozymes with peroxidase-like activity. BSA-Cu SAN efficiently catalyzed H_2_O_2_ to generate ROS and deplete GSH simultaneously, leading to successful scavenging of *F. nucleatum* in situ, effectively restoring the autophagy level of tumor cells elevated by *F. nucleatum*, relieving ROS resistance, and bringing about intracellular redox imbalance and mitochondrial dysfunction for apoptosis of colorectal cancer cells. Furthermore, BSA-Cu SAN can be thoroughly cleared by the kidney, exempting it from long-term systemic toxicity, which is indispensable progress for the clinical transformation of SANs. This study provides a promising pathogen-focused cancer therapy by destroying pathogen-tumor symbionts which are not commonly utilized as therapeutic targets, and an appreciated trial of constructing highly-biomimetic artificial protein-supported single-atom nanozymes for the process of clinical transformation of SAN catalytic therapy.

## Materials and methods

### Preparation of BSA-Cu SAN

BSA (500 mg) was dissolved in 250 ml double distilled water. CuCl_2_ (53.6 mg) in 5 ml water solution was dropped in and stirred at room temperature. After 30 min, 2 M NaOH solution (2.4 ml) was rapidly added to the mixture to reach the pH value of 12, and the solution turned from light blue to dark purple instantly. After another 30 min at 78 °C, 264.2 mg of ascorbic acid was added to the above solution and stirred for 1 h at 78 °C. During that time, the solution turned transparent brown gradually. Finally, BSA-Cu SAN was collected after washing and concentrating through centrifugation with 10 KD ultrafiltration membranes.

### Catalytic function of BSA-Cu SAN

The production of •OH was detected by ESR spectroscopy. In total, 80 μl of DMPO (100 mM) was added as a spin trap to 40 μl of BSA-Cu SAN solution containing 30 μg/ml of Cu with or without 1 mM of H_2_O_2_ and then conducted on the EMXplus-6/1 spectrometer. TMB assay confirmed ROS generation. Distilled water, BSA-Cu SAN (30 μg/ml Cu), H_2_O_2_ (1 mM), and BSA-Cu SAN + H_2_O_2_ were added to the TMB solution (1 mM) to make the final volume of 5 ml. The color changes of the solution were recorded by photos and UV-vis absorption spectrum in different response times. PBS at pH 5.5, 6.5, and 7.4 were utilized to study the effect of acidity or basicity on the catalytic reaction. Furthermore, 30 μg/ml Cu of BSA-Cu SAN and 0.2–2 mM of H_2_O_2_ were added to the TMB solution at pH 6.5 to analyze the catalytic velocity further. The UV-vis absorption at 652 nm was recorded every 2 s for 5 min and calculated by the Michaelis–Menten equation:

$$\begin{array}{ll}v_0 = {{{{V}}}}_{{{{\mathrm{max}}}}} \cdot \left[ {{{\rm{S}}}} \right]/\left( {{{{{K}}}}_{{{\mathrm{m}}}} + \left[ {{{\mathrm{S}}}} \right]} \right)\\ \frac{1}{{v_0}} = \frac{{{{{\mathrm{km}}}}}}{{{{{{V}}}}_{\max }}} \cdot \frac{1}{{\left[ {{{\mathrm{S}}}} \right]}} + \frac{1}{{{{{{V}}}}_{\max }}}\end{array}$$

### GSH consuming detection

BSA-Cu SAN containing 15, 30 or 45 μg/ml Cu was mixed with GSH solution (1 mM) and reacted for different times. At certain time, 100 μl solution was taken out and diluted ten times with PBS. Then 10 μl DTNB (10 mM) was added, and the absorbance spectrum of the solution was detected by UV-vis spectrophotometer.

### Antibacterial assay in vitro

*F. nucleatum* in the logarithmic growth phase was divided into the control and the BSA-Cu SAN antibacterial test. In the test tube, *F. nucleatum* was incubated with 45 μg/ml BSA-Cu SAN, and all the tubes containing *F. nucleatum* were incubated at 37 °C in the anaerobic chamber. At various times of 6, 12, 18, 24, 32, 42, 54, and 72 h, 100 μl bacterial suspension was quickly removed, diluted 10^6^ times with saline, and spread on the Columbia blood agar plates. The plates were cultured at 37 °C in an anaerobic chamber for 72 h, and the bacterial colonies were calculated by ImageJ (National Institute of Health, USA). The time-inhibition curve was drawn according to the number of colonies.

*F. nucleatum* in the logarithmic growth phase were incubated with 100 μM H_2_O_2_, 45 μg/ml BSA-Cu SAN or BSA-Cu SAN + H_2_O_2_ to find the catalytic function of BSA-Cu SAN in antibacterial more intuitively. After 6 h in an anaerobic atmosphere, 100 μl suspension in each tube was extracted and diluted 10^3^ times before spreading on the Columbia blood agar plates. In addition, SEM observed the destruction of the specific morphology of *F. nucleatum*. *F. nucleatum* suspensions were also centrifugated and re-suspended with saline solution. Live & Dead Bacterial Staining Kit was utilized to identify the viability of *F. nucleatum* under different treatments. DMAO and EthD-III were used in the kit to stain bacteria for 15 min in the dark. Finally, a 5 μl mixture was dropped on the glass slide for observation under CLSM. *F. nucleatum* suspension was stained with DCFH-DA for 30 min and then read by the microplate reader at *λ*_ex_ = 488 nm and *λ*_em_ = 525 nm to detect the generation of ROS.

### Cell proliferation and cytotoxicity assays in vitro

Cells were seeded in the 96-well plates at a density of 6 × 10^3^ cells per well. The previous medium was removed overnight, and 6 × 10^5^ CFU/ml *F. nucleatum* was re-suspended in 100 μl DMEM to the plates. After 12, 24, and 48 h, the cells were lightly washed with PBS twice, and 10% CCK-8 was added to the plates. Finally, the absorption of 450 nm was detected via a microplate reader. For cytotoxicity assay, HCT116 cells infected by *F. nucleatum* were treated with various concentrations of BSA-Cu SAN with or without 100 μM H_2_O_2_ under pH 6.5 or 7.4. After 24 h of incubation, cells were washed with PBS twice, put in 10% CCK-8, and detected by the microplate reader. Cell viability was calculated using Eq. (1):

1 $$\begin{array}{l}{\rm{Cell}}\,{\rm{viability}}\,\left( \% \right) = \\\left( {{\rm{OD}}_{450{\rm{nm}}/{\rm{sample}}} - {\rm{OD}}_{450{\rm{nm}}/{\rm{sample}}}} \right)/\left( {{\rm{OD}}_{450{\rm{nm}}/{\rm{control}}} - {\rm{OD}}_{450{\rm{nm}}/{\rm{blank}}}} \right) \times 100\%\end{array}$$

### Live/dead cell staining assay

HCT116 cells were seeded in confocal laser dishes at a density of 1 × 10^4^ per dish. After 24 h of cell adhesion, the medium was discarded and substituted for fresh medium containing various samples (45 μg/ml BSA-Cu SAN, 100 μM H_2_O_2,_ or BSA-Cu SAN + H_2_O_2_) under pH 6.5. The confocal laser dishes were incubated for another 24 h at 37 °C. Then cells were dyed with Calcein-AM/PI for 15 min and viewed via CLSM.

### Mitochondrial potential assay

An enhanced mitochondrial membrane potential assay kit with JC-1 detected cell apoptosis. After growing in the confocal laser dishes overnight, HCT116 cells were incubated with BSA-Cu SAN with or without H_2_O_2_ for 24 h. Subsequently, cells were stained with JC-1 in its working concentration according to the protocol for 20 min at 37 °C and then washed twice with the provided buffer. CLSM observed the fluorescence images.

### Flow cytometry experiments for apoptosis assay

HCT116 cells seeded in 6-well plates were cultured overnight in the incubator for adhesion. They were given the same samples as a potential mitochondrial assay. After 24 h of incubation, all cells were collected by trypsin without EDTA, washed twice with PBS, and stained with Annexin V-FITC/PI in the dark for 15 min at room temperature. Lastly, the cells were subjected to the flow cytometer and analyzed by FlowJo software (BD Bioscience, USA).

### Western blot analysis

HCT116 cells were harvested after different treatments for 24 h. RIPA lysis buffer containing protease inhibitor was added to the cells for lysis. After quantification of protein concentration by the BCA method, the protein samples were mixed with loading buffer and boiled at 95 °C for 10 min to efficiently denature the protein. Then the protein samples were isolated by 12.5% SDS-PAGE at 80 V and transferred to 0.22 μm PVDF membrane at 200 mA for 30 min. The membrane was primarily blocked with the protein-free rapid-blocking buffer for 15 min at room temperature. GAPDH (1:1000) and LC3 (1:1000) antibodies were incubated overnight at 4 °C, then with the secondary antibody (1:5000) for 1 h at room temperature after washing with TBST. Finally, the ECL system was utilized to visualize the blot bands.

### Animal experiments

All mice treatments obeyed the rules of the Ethics Committee of Shanghai Tenth People’s Hospital, affiliated with Tongji University. This study purchased 4 weeks old male BALB/c nude mice and BLAB/c mice from Shanghai Sippe-Bk Lab Animal Co., Ltd. They were bred in the specified pathogen-free (SPF) animal barrier (Science and Technology Innovation Park, Shanghai Tenth People’s Hospital, Shanghai, China). Animals were kept in 22 ± 5 °C and 55 ± 15% humidity rooms. Sufficient space for movement and a plentiful supply of food and drinking water was provided in the cages.

### Evaluation of biodistribution and clearance

Hypodermic injections of 1 × 10^6^ HCT116 cells were conducted to the left inguinal regions of BALB/c nude mice aged 5 weeks. When the tumor size was ~50 cm^3^, 4 mg/kg BSA-Cu-Cy5.5 was injected intravenously into each mouse. Blood was taken out at different time points for fluorescence, and exact Cu contents were measured by ICP. Mice were sacrificed at specific times of 0, 0.5, 2, 8, 12, 24, and 48 h. The vital organs and tumors were dissected for fluorescence imaging.

### Statistic methods

The significance of the data was analyzed according to one-way ANOVA or Student’s *t* test method: *p < 0.05, **p < 0.01, ***p < 0.001, and ****p < 0.0001, respectively. Data were presented as the mean ± SD. The samples/animals were allocated to experimental groups and processed randomly.

## Supplementary information

SUPPLEMENTAL MATERIAL

## Data Availability

The raw/processed data required to reproduce these findings are available upon reasonable request to corresponding authors.
