# Supplementary material for: Destroying pathogen-tumor symbionts synergizing with catalytic therapy of colorectal cancer by biomimetic protein-supported single-atom nanozyme
Source: Signal Transduct Target Ther. 2023 Jul 21;8:277. doi: 10.1038/s41392-023-01491-8 (PMC10359331; doi:10.1038/s41392-023-01491-8)
Supplement: Supplementary file 1 — SUPPLEMENTAL MATERIAL [file 41392_2023_1491_MOESM1_ESM.docx]

Supplementary Materials for

Destroying Pathogen-Tumor Symbionts Synergizing with Catalytic Therapy of Colorectal Cancer by Biomimetic Protein-supported Single-Atom Nanozyme

Xinyue Wang^1^, Qian Chen^1,^ *, Yefei Zhu^1^, Kairuo Wang^1^, Yongliang Chang^1^, Xiawei Wu^1^, Weichao Bao^3^, Tongcheng Cao^4^, Hangrong Chen^3^, Yang Zhang^1,^ ^2,^ *, Huanlong Qin^1,^ *

Correspondence to:

chenqian163329@163.com, [zhangyang0202@tongji.edu.cn](mailto:zhangyang0202@tongji.edu.cn), hlongqin@126.com

**This PDF file includes:**

Materials and Methods

Figures. S1 to S28

Tables S1 to S4

Materials and Methods

Materials and reagents

Copper chloride (CuCl_2_), sodium hydroxide (NaOH), hydrogen peroxide aqueous solution (H_2_O_2_), reduced glutathione (GSH), and DTNB were purchased from Aladdin Co., Ltd. Bovine serum albumin (BSA) was purchased from MP Biomedicals (Shanghai) Co., Ltd. Dulbecco’s minimal essential medium (DMEM), fetal bovine serum (FBS) and streptomycin/penicillin were bought from Gibco, CA. L-Ascorbic acid and 3,3′,5,5′- tetramethylbenzidine dihydrochloride hydrate (TMB) was purchased from BBI CO., LTD. Cell Counting Kit-8, Annexin V FITC/PI Apoptosis Detection Kit, Cell Cycle Kit, Live & Dead Bacterial Staining Kit, and β-actin (Cat. #30101ES10) were purchased from Yeasen Biotechnology (Shanghai) Co., Ltd. Hoechst 33258, DCFH-DA and JC-1 MitoMP detection kit were obtained from Beyotime Biotechnology Co., Ltd. WGA594 was provided by Invitrogen. Reagents for WB were all purchased from Shanghai Epizyme Biomedical Technology Co., Ltd. LC3A/B (D3U4C) XP Rabbit mAb (Cat. #12741T) was purchased from Cell Signaling Technology, and GAPDH, rabbit pAb was purchased from Yeasen Biotechnology (Shanghai) Co., Ltd. HCT116, NCM460, HK-2 cells and Fusobacterium nucleatum (ATCC 25586) were purchased from the American Type Culture Collection (ATCC). Thioglycollate Medium was bought from Qingdao Hope Bio-Technology Co., Ltd., China.

Instruments

Confocal microscopy images were taken by CarlZeiss LSM900, and florescent images of ROS were taken by Nikon TS2R-C-AL microscope. Flow cytometry was performed on a BD fortessa cytometer. Cell viability and fluorescence were detected by a microplate reader (Molecular Devices SpectraMax iD5, China).

Experiment methods

Characterization of BSA-Cu SAN: The morphology of BSA-Cu SAN was observed by transmission electron microscope (TEM, FEI Tecnai G2 F20, USA). The samples’ atomic structures were observed by an aberration-corrected scanning transmission electron microscope (AC-STEM, Hitachi HF5000, Japan) equipped with an energy-dispersive X-ray spectroscopy system (EDS, Oxford X-Max, UK). Hitachi SU8010 obtained SEM images. The UV-vis and XPS spectra were performed by UV-vis spectroscopy (Shinamadzu UV-2600i) and Thermo Scientific K-Alpha, respectively. The concentration of Cu was measured by ICP-OES (Agilent 5110), and the ESR signal was detected by Bruker EMX PLUS-6/1 (Bruker, Germany). DLS and zeta potential were conducted by Zetasizer Lab-Blue (MAL1265848) instrument. Thermo Scientific Nicolet iS20 scanned FT-IR spectra.

Preparation of Cy5.5-labeled BSA-Cu SAN: Cy5.5-labeled BSA-Cu SAN was prepared according to previous references^48^. In this study, BSA-Cu SANs (4 mL, 1.5 mg/mL) were mixed with Cy5.5-NHS (1 mL, 0.1 mg/mL), and the mixture was stirring at room temperature for 8 hours away from the light. After that, the mixed solution was washed by water to remove unconjugated Cy5.5, and concentrated through ultrafiltration. Fluorescence spectra was used to characterize Cy5.5-labeled BSA-Cu SAN (λ_ex_ = 670 nm, λ_em_ = 695 nm).

Quantum mechanical calculation：Gaussian 16 package was used to conduct quantum mechanical calculations. Geometries of intermediates and transition states were optimized by the B3LYP-D3 functional 2-3 (with Grimme’s D3 dispersion) with a mixed basis set (BS1) of SDD for Cu and 6-31G(d,p) for other atoms in the gas phase. Singlet-point energies were calculated at the same level of theory as the geometry optimization using the SMD solvation model4 with ε = 4.0 to estimate the surrounding effects. At the same level as geometry optimization, frequency calculations were performed to obtain zero-point energies (ZPE). To get more accurate energies, single-point calculations on the optimized structures were performed with the larger mixed basis set (BS2) of SDD for Cu and 6-311+G(2d,2p) for other atoms. The presented values are thus the BS2 energies, corrected for ZPE and solvation effects.

Preparation of Fusobacterium nucleatum suspension: *F. nucleatum* was revived from frozen stocks by inoculation in a hioglycolate medium. The procedure was done overnight at 37 ℃ in the anaerobic chamber to keep bacteria in the logarithmic growth phase. Then, *F. nucleatum* was collected by centrifugation at 8000 rpm for 3 min and re-suspended with sterile saline. The population density of the bacterial cells was determined by measuring the absorbance at 600 nm.

Cell culture: HCT116 cell lines, NCM460 cells, and HK-2 cells were grown in DMEM with 10% FBS and 1% streptomycin/Penicillin. All cells were cultured in a humidified atmosphere at 37 ℃ and 5% CO_2_ in a standard cell incubator (Thermo 3111, USA).

Cellular uptake evaluation: HCT116 cells were seeded in confocal laser dishes or a 6-well plate. After 24 h of cell adhesion, the medium was discarded and substituted for fresh medium containing 30 μg/mL BSA-Cu-FITC. The mixture was incubated for 2, 4, 6, and 8 h at 37 ℃. For fluorescence images, Hoechst 33258 for nucleus staining and WGA594 membrane staining were added to the dishes in sequence for 10 min. The cells were washed in PBS twice and viewed by CLSM. The cells were collected in tubes and subjected to flow cytometry analysis for precise quantity.

ROS detection in vitro: HCT116 cells were seeded in a 12-well plate and incubated for 24 h for cell adhesion. The medium was removed, and fresh medium containing different samples was added to the wells (control, 100 μM H_2_O_2_, 30 μg/mL BSA-Cu SAN with or without H_2_O_2_) and incubated at 37 ℃ with 5% CO_2_ atmosphere for 6 h based on the result of cellular uptake evaluation. The culture medium’s pH value was modified to 6.5 using HCl to simulate the tumor microenvironment. The following steps were conducted according to the DCFH-DA manufacturer’s protocol. The medium was removed, the cells washed with PBS, diluted DCFH-DA (1:1000) to working concentration, and then added to the plate. After incubating for 30 min at 37 ℃ in the dark field, cells were washed in PBS and viewed via a fluorescence microscope.

Histology analysis: After each group’s organs and tumor tissues were collected and photographed, they were fixed in 4% paraformaldehyde for 24 h at least. Increasing concentrations of alcohol were used for dehydration before embedding paraffin and slicing it into 5 mm thick pieces. The following procedures were guided by the standard protocol, such as deparaffinization, hydration, and staining with H&E. TUNEL staining and ki67 staining complied with the manufacturer’s instructions.

Figure. S1.


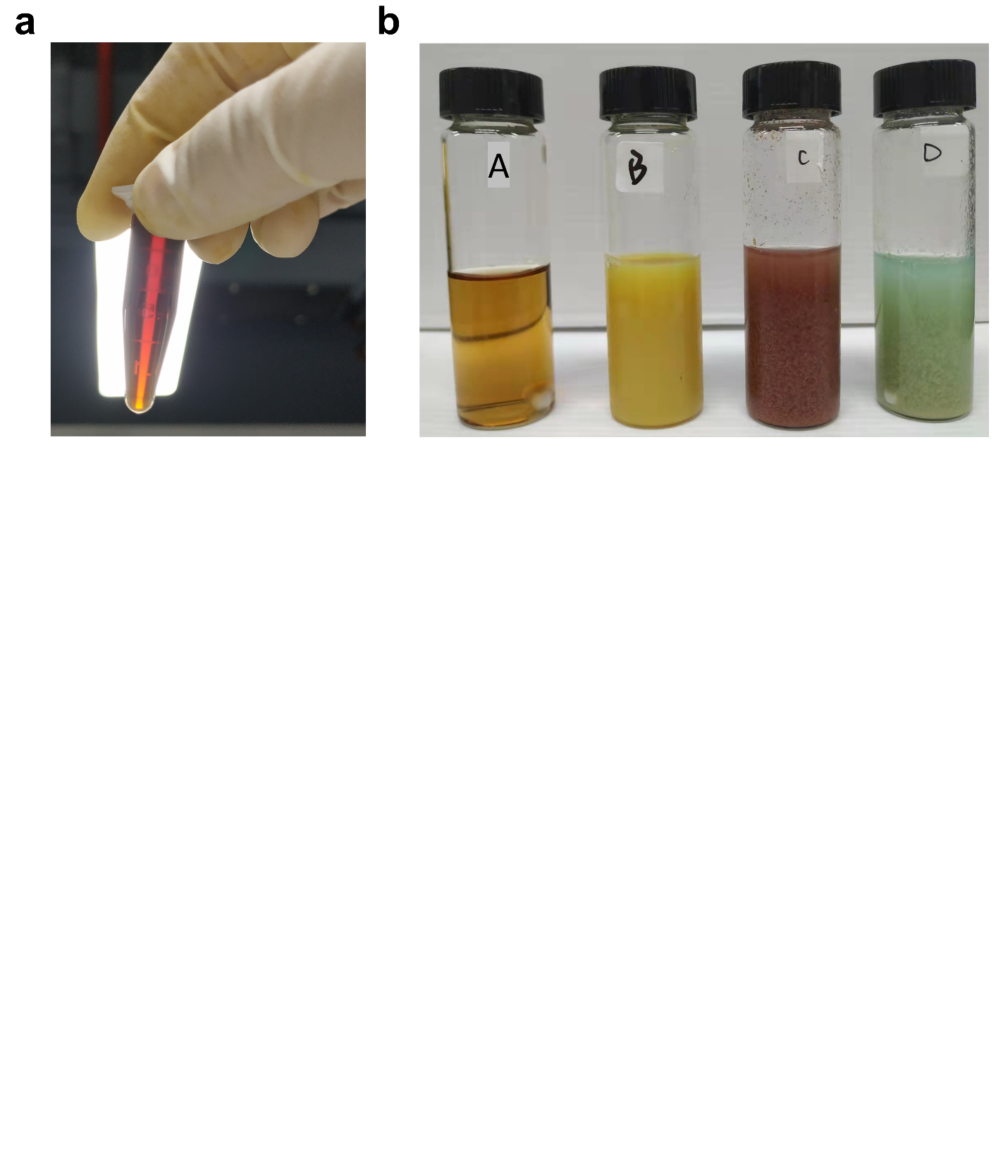


**Figure. S1. The photos of the products with different Cu contents.** (a) The photograph of BSA-Cu SAN after ultrafiltration. (b) The photograph of different end-products obtained after adding different amounts of CuCl_2_. A-D represent copper loading at 5.42 wt% (BSA-Cu SANs), 10.8 wt%, 21.7 wt%, and 43.4 wt%, respectively.

Figure. S2.


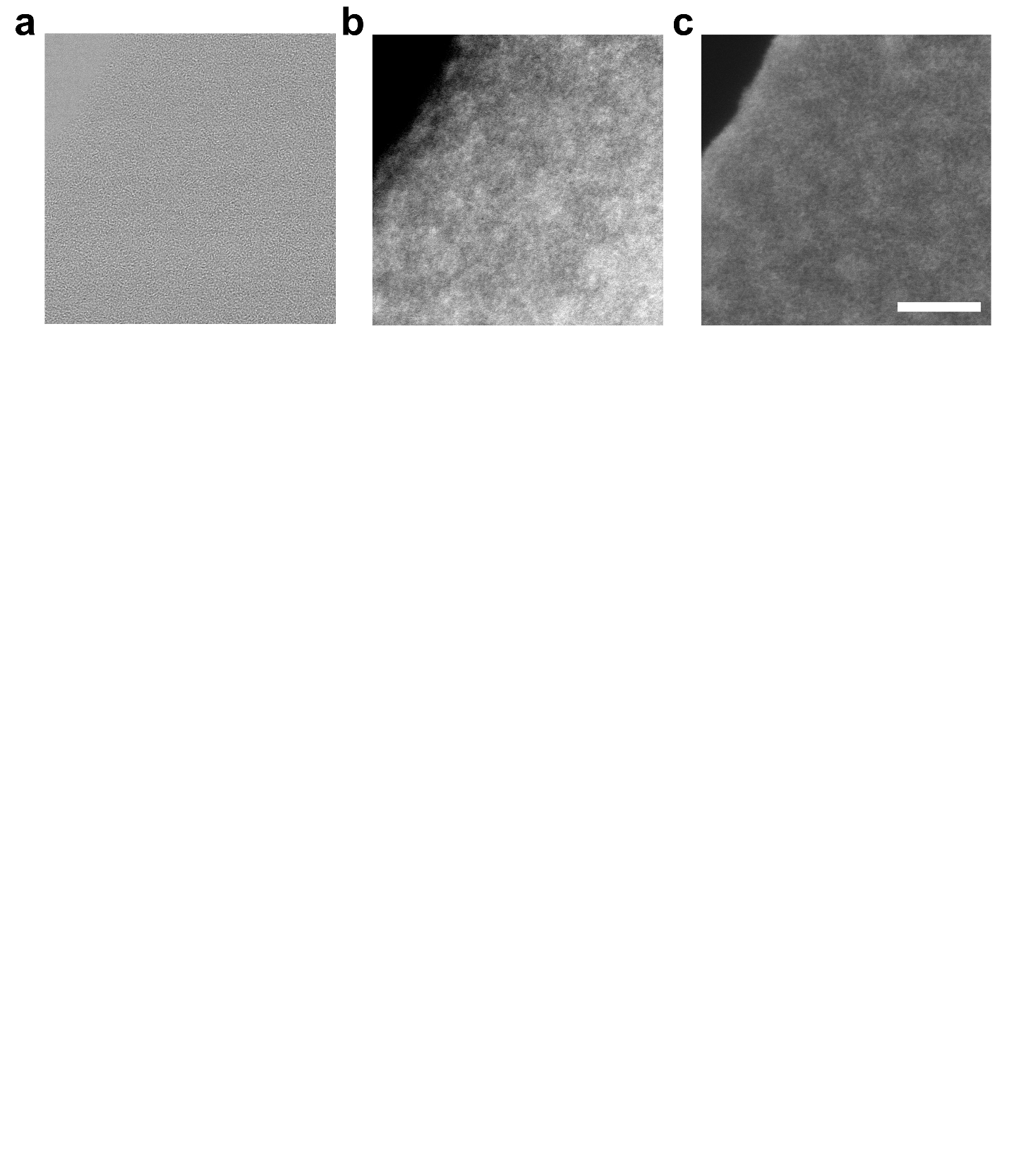


**Figure. S2.** TEM images of BSA-Cu SAN. (a) Bright field (b) HAADF (c) Secondary electrons. Scale bar = 10 nm.

Figure. S3.


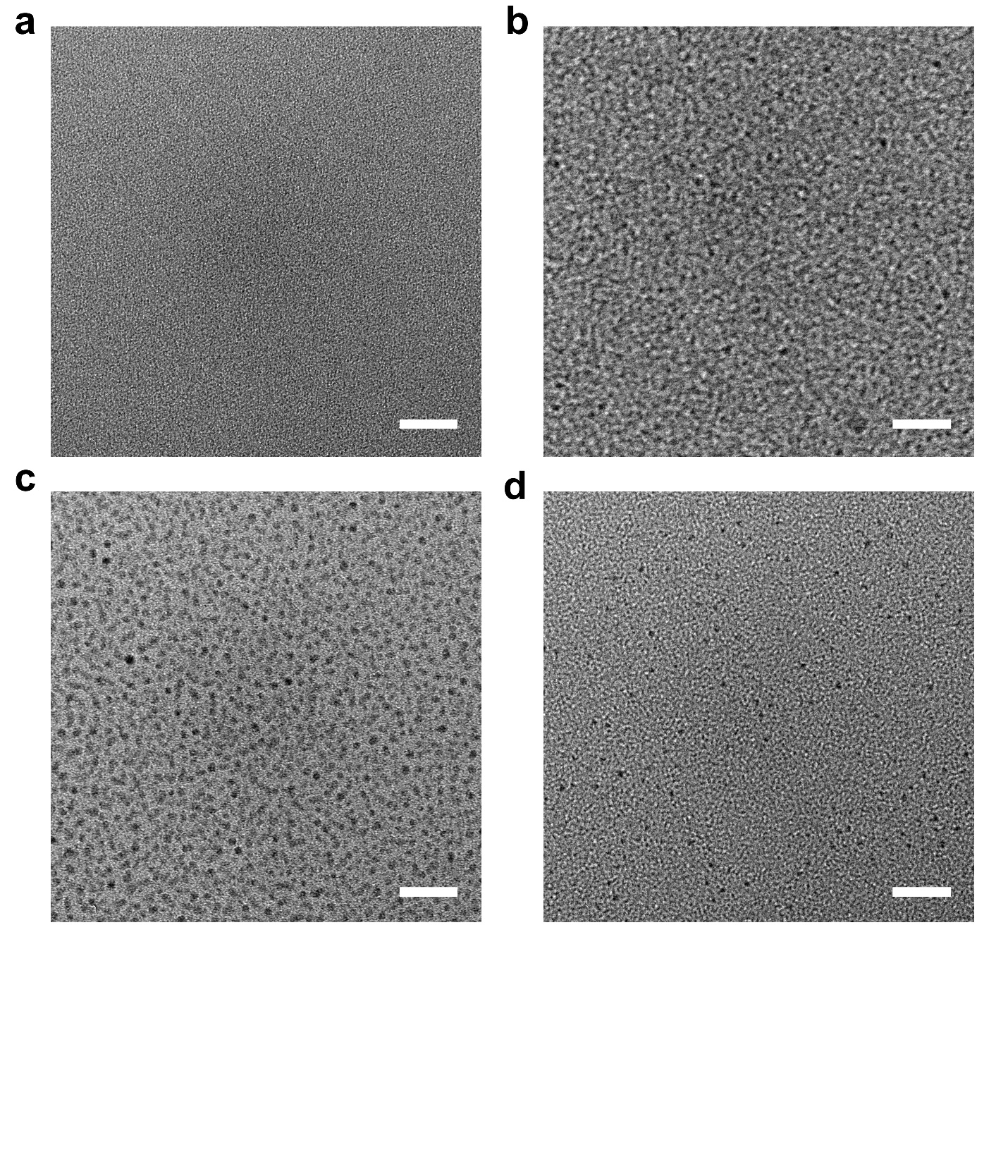


**Figure. S3.** TEM images of the end products with different input of CuCl_2_. (a) BSA-Cu SAN with copper loading at 5.42wt%. (b) 2-fold amount of CuCl_2_ of (a). (c) 4-fold amount of CuCl_2_ of (a). (d) 8-fold amount of CuCl_2_ of (a). Scale bar = 20 nm.

Figure. S4.


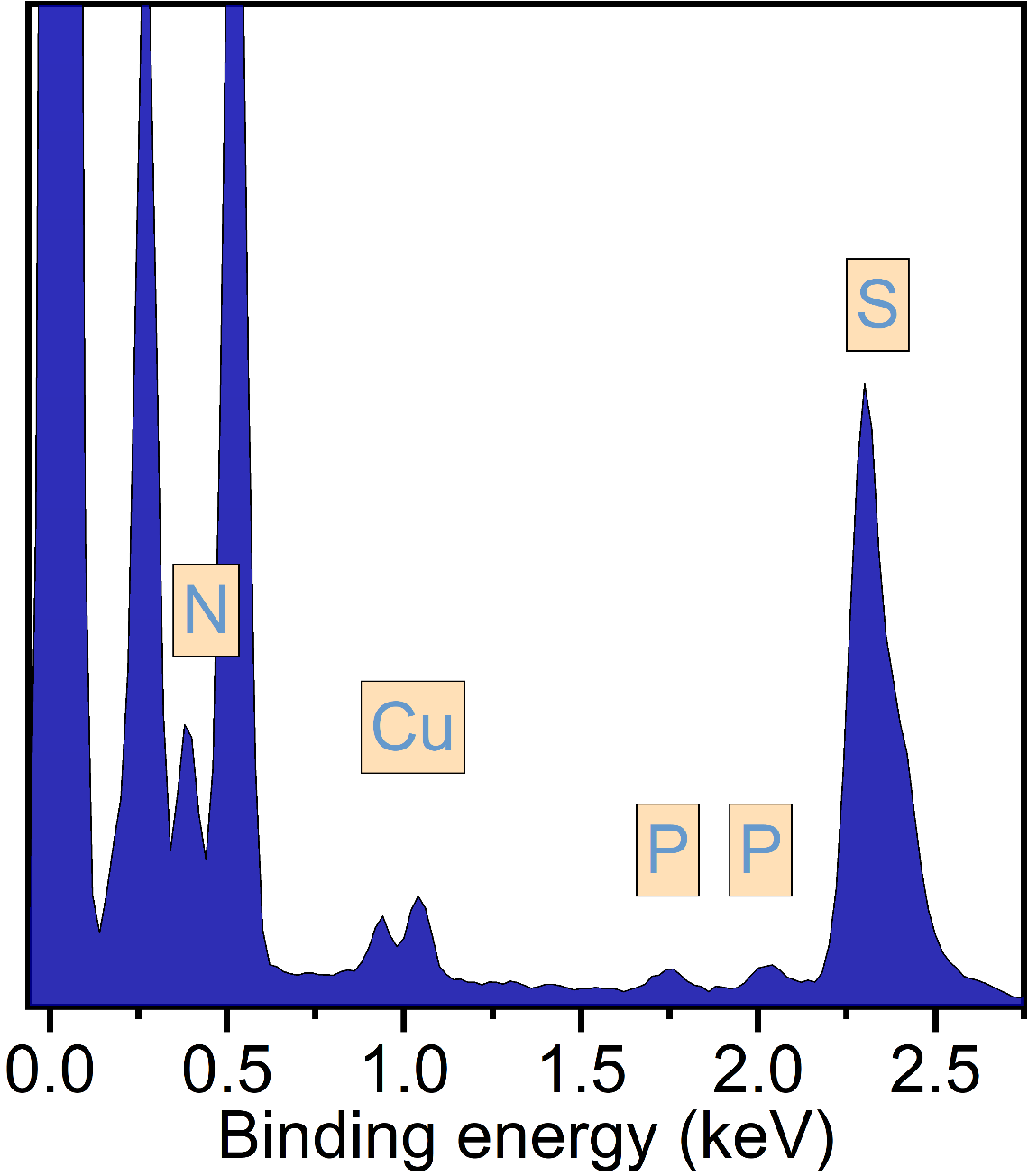


**Figure. S4.** The corresponding EDS of BSA-Cu SAN.

Figure. S5.


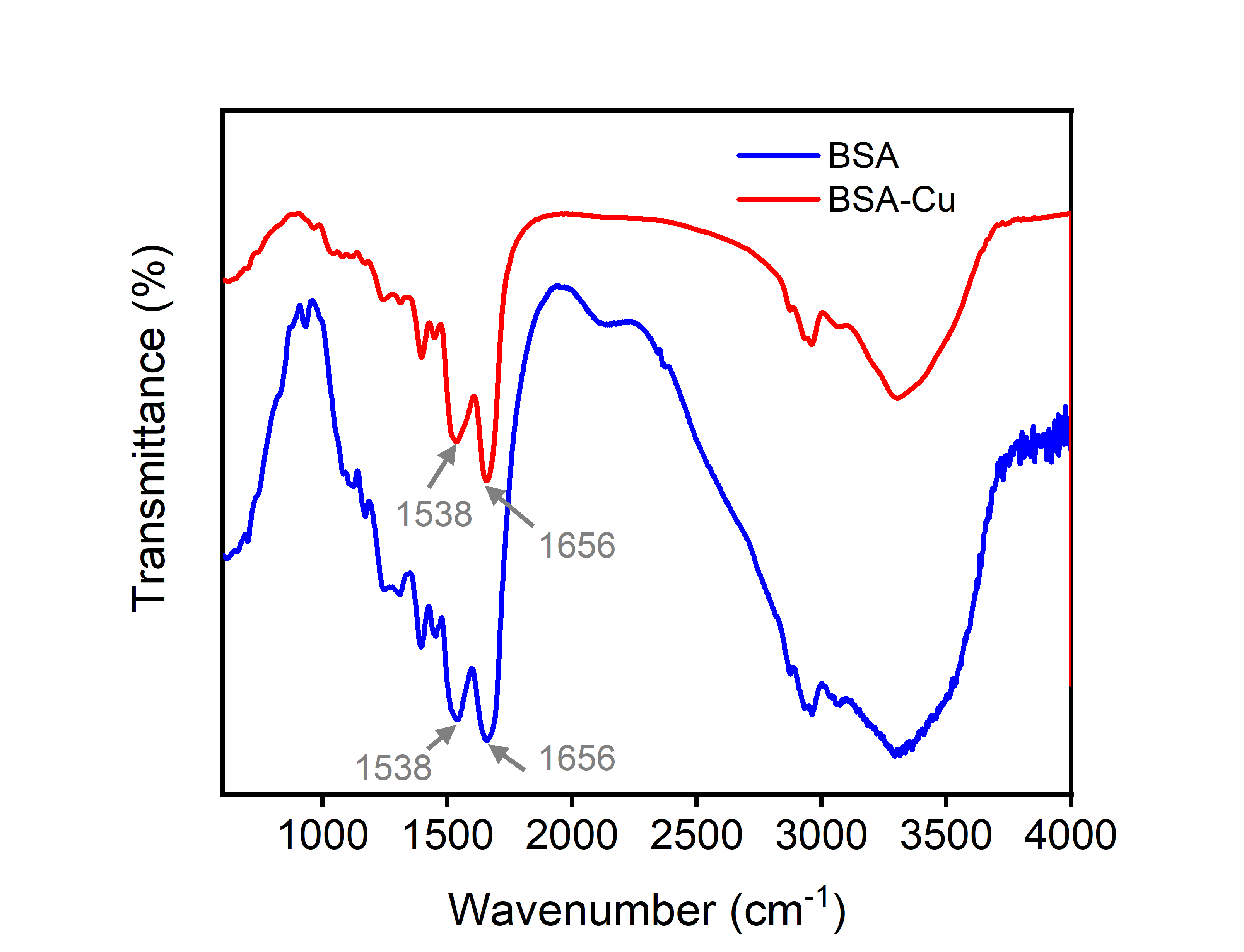


**Figure. S5.** FT-IR spectra of BSA and BSA-Cu SAN.

Figure. S6.


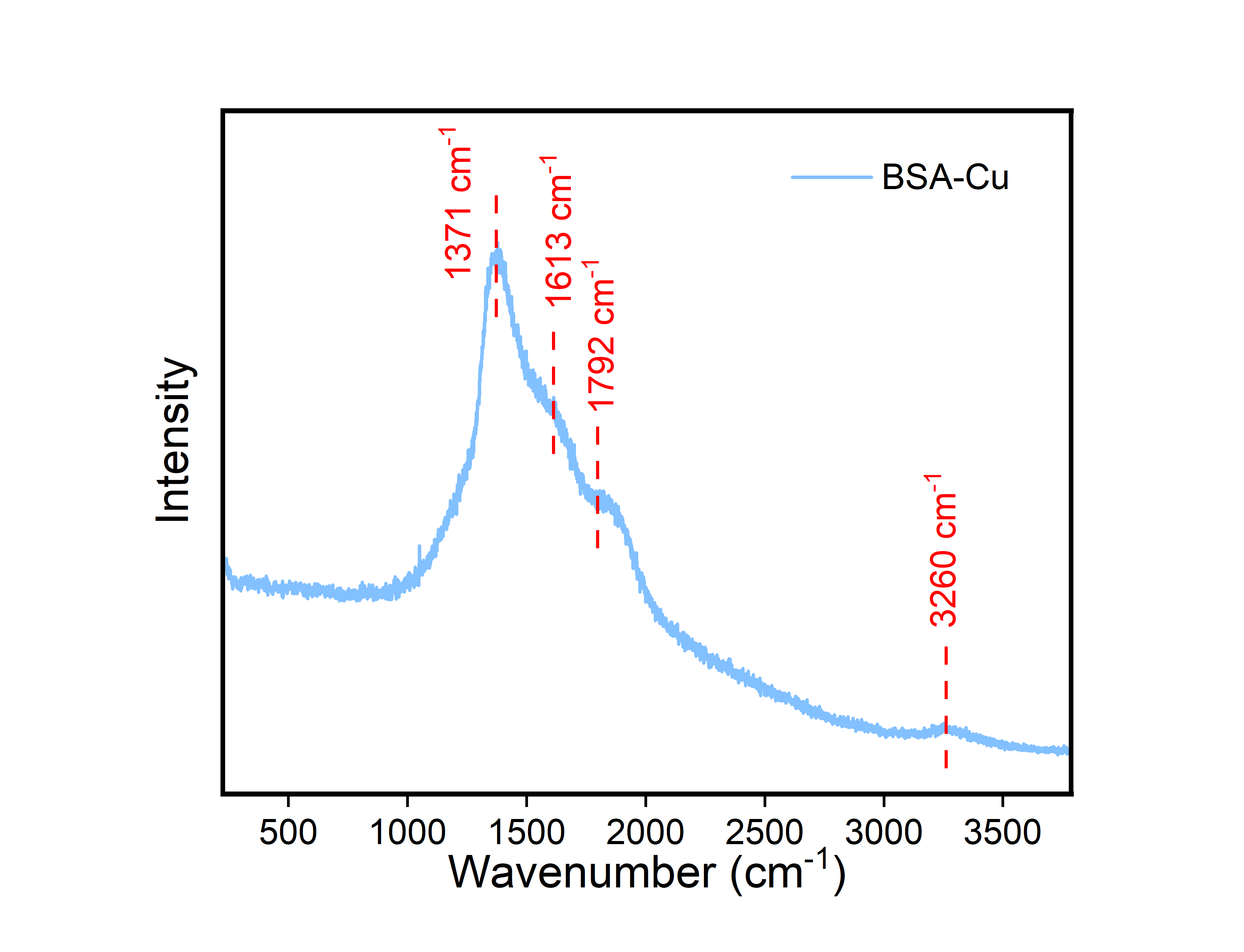


**Figure. S6.** Raman spectra of BSA-Cu SAN. 3260 cm^-1^ is the stretching vibration of NH. 1792 cm^-1^ is the stretching vibration of C=O (amide characteristic peak I), and 1613 cm^-1^ is the amide characteristic peak II, which comes from the coupling of C-N stretching vibration and NH bending vibration, with NH bending vibration as the main contribution. 1371 cm^-1^ is the characteristic peak III of the amide that comes from the coupling of the C-N stretching vibration and the NH bending vibration, and the C-N stretching vibration is the main contribution. The above Raman spectrum confirms the existence of an amide structure in the structure by identifying the characteristic peaks of the amide.

Figure. S7.


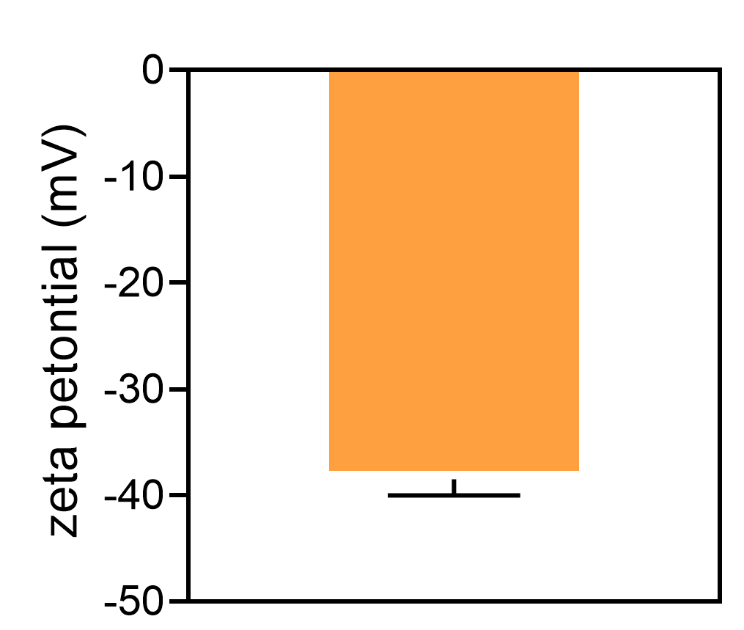


**Figure. S7.** Zeta potential of BSA-Cu SAN.

Figure. S8.


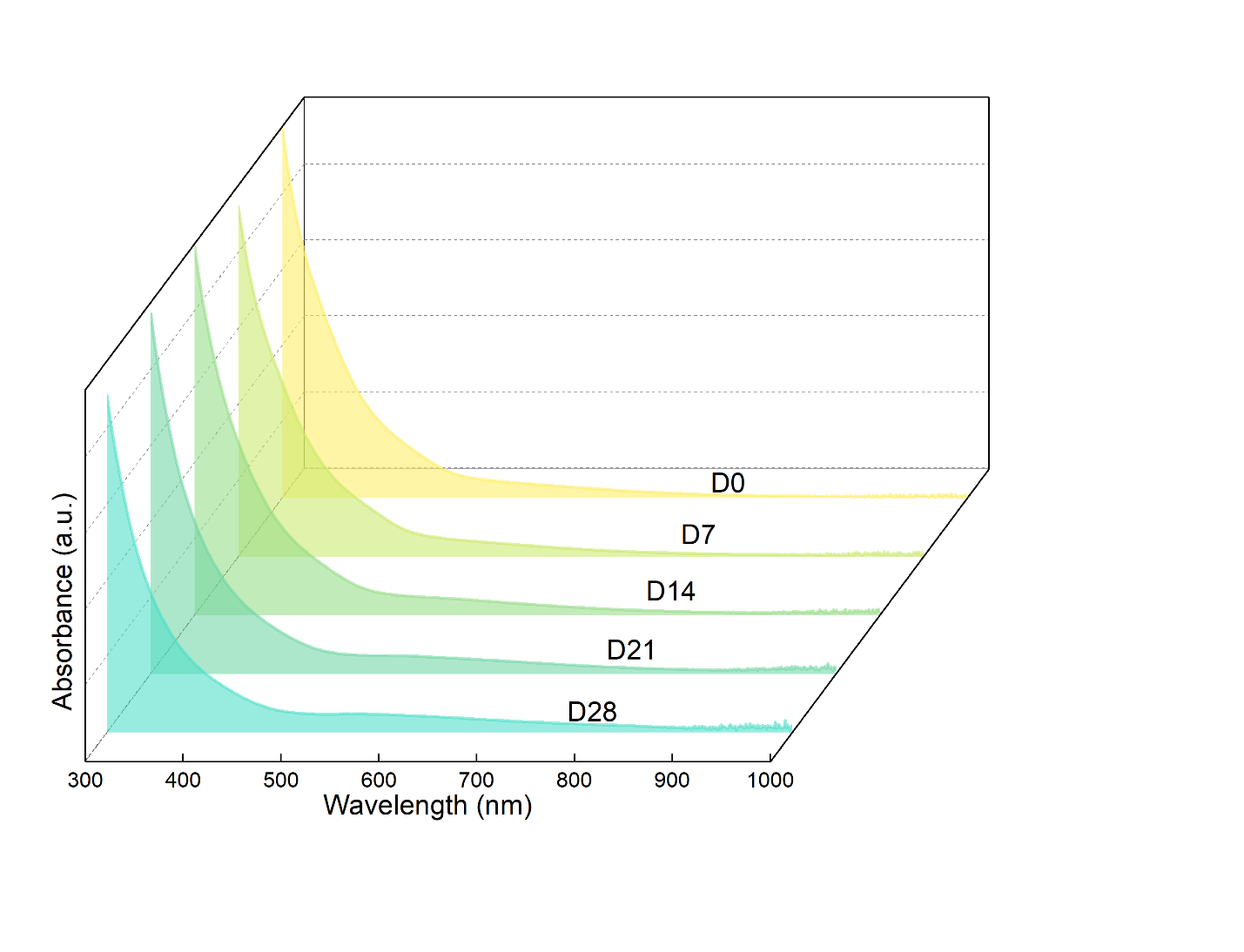


**Figure. S8.** UV absorption spectra of the same sample of BSA-Cu SANs measured every week for 4 weeks.

Figure. S9.


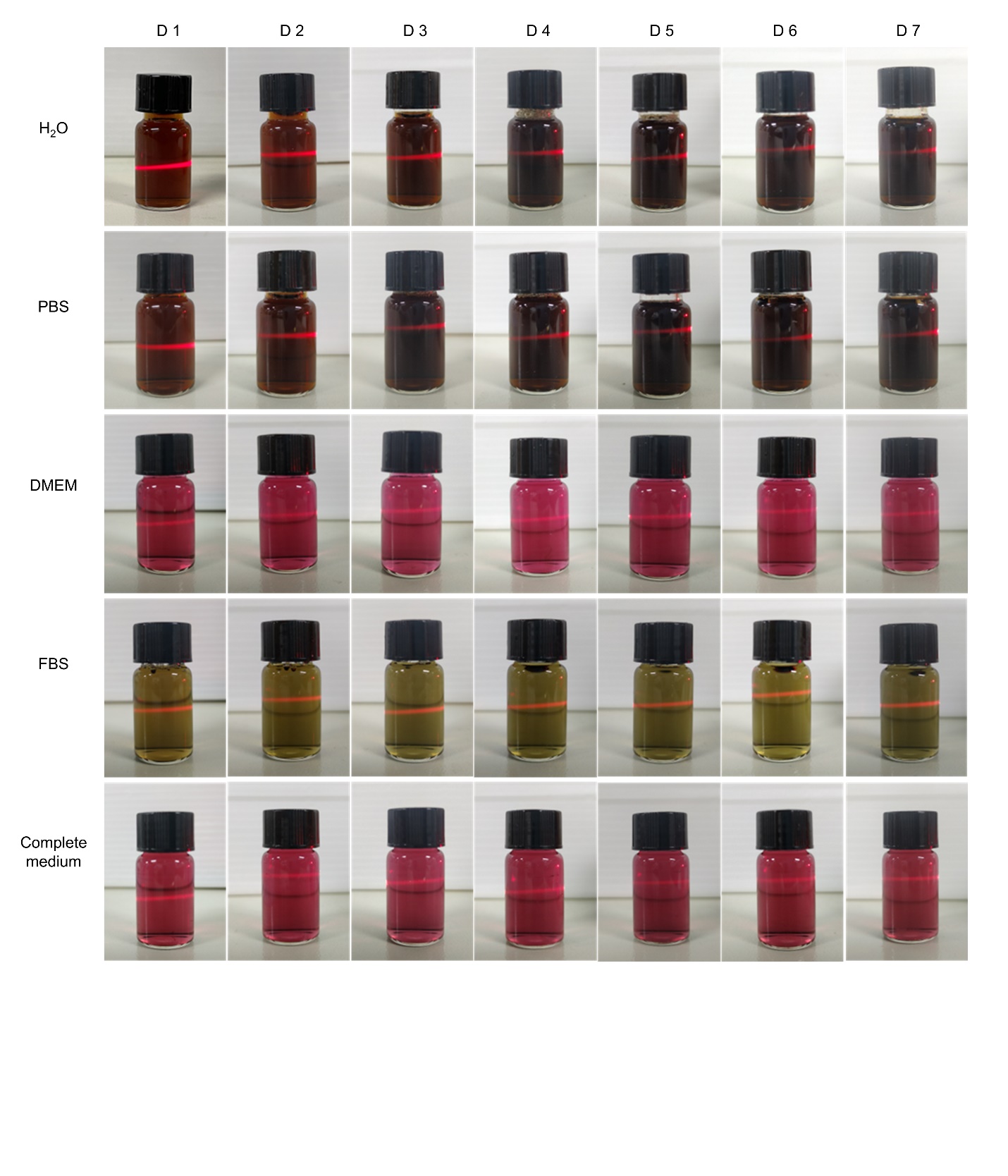


**Figure. S9.** The photos of BSA-Cu SAN in water, PBS, DMEM, FBS and complete medium for seven days. The red light beam is launched by a laser pointer.

Figure. S10.


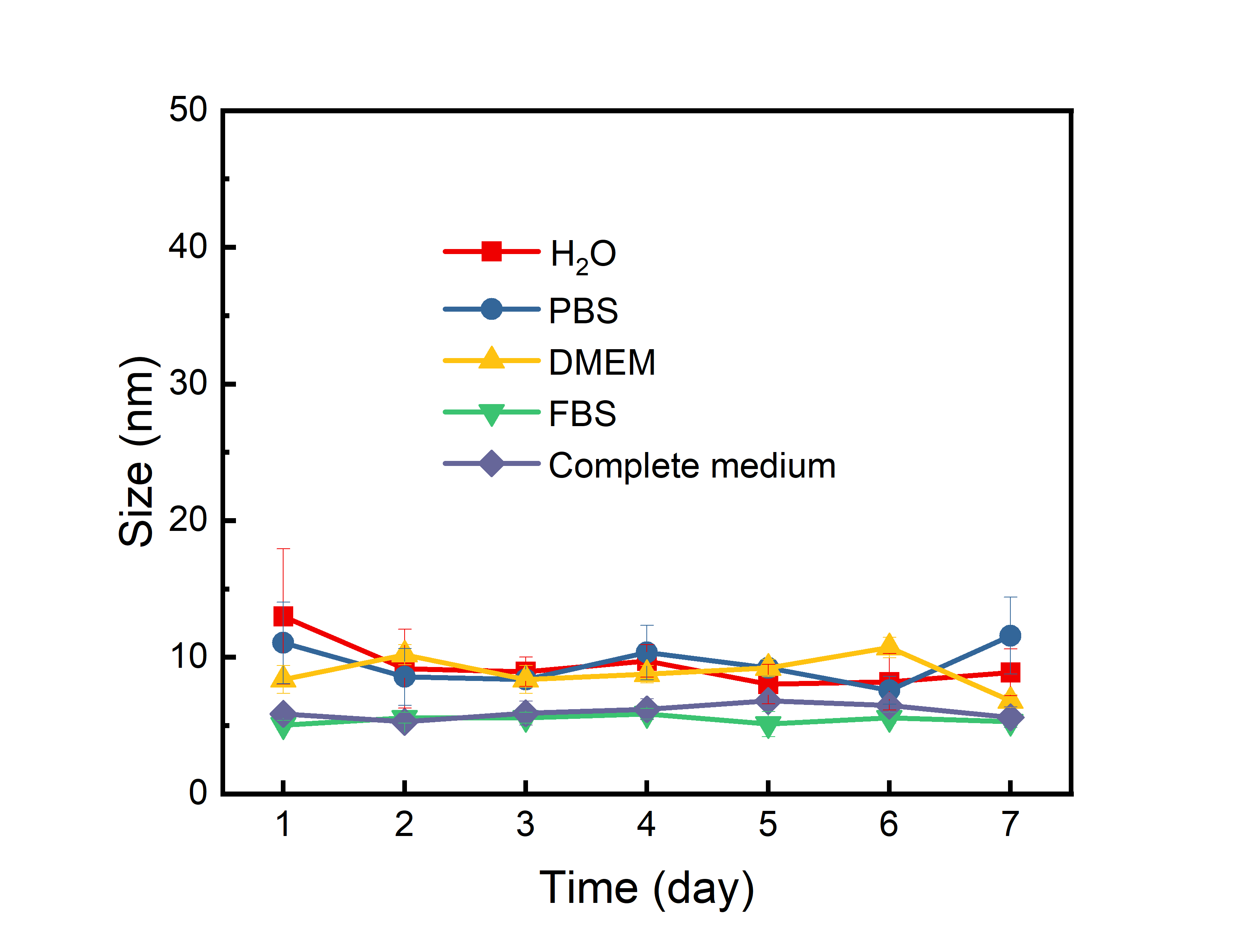


**Figure. S10.** Dynamic light scattering of BSA-Cu SAN in water, PBS, DMEM, FBS and complete medium for seven days.

Figure. S11.


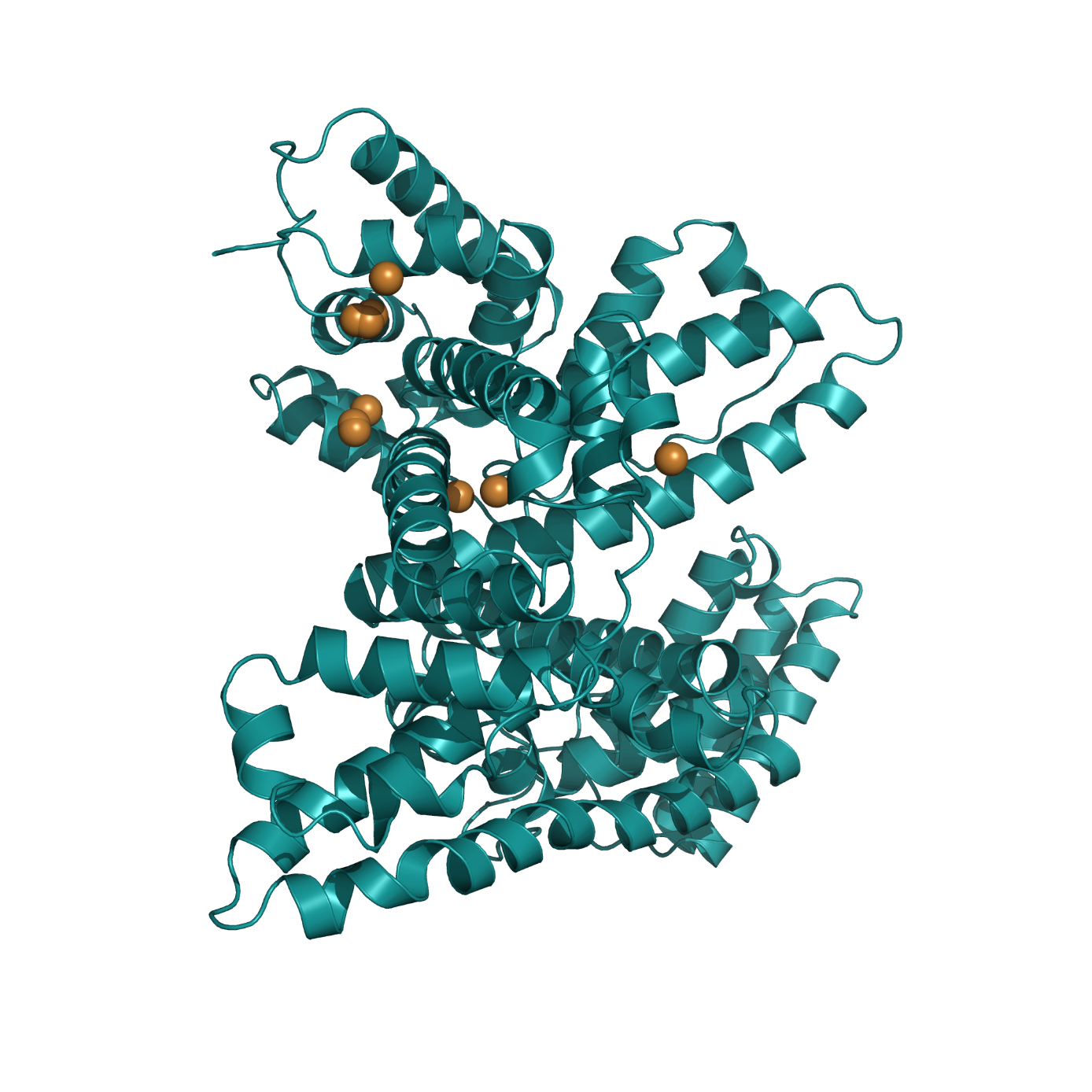


**Figure. S11.** The potential binding sites of Cu^+^ on BSA.

Figure. S12.


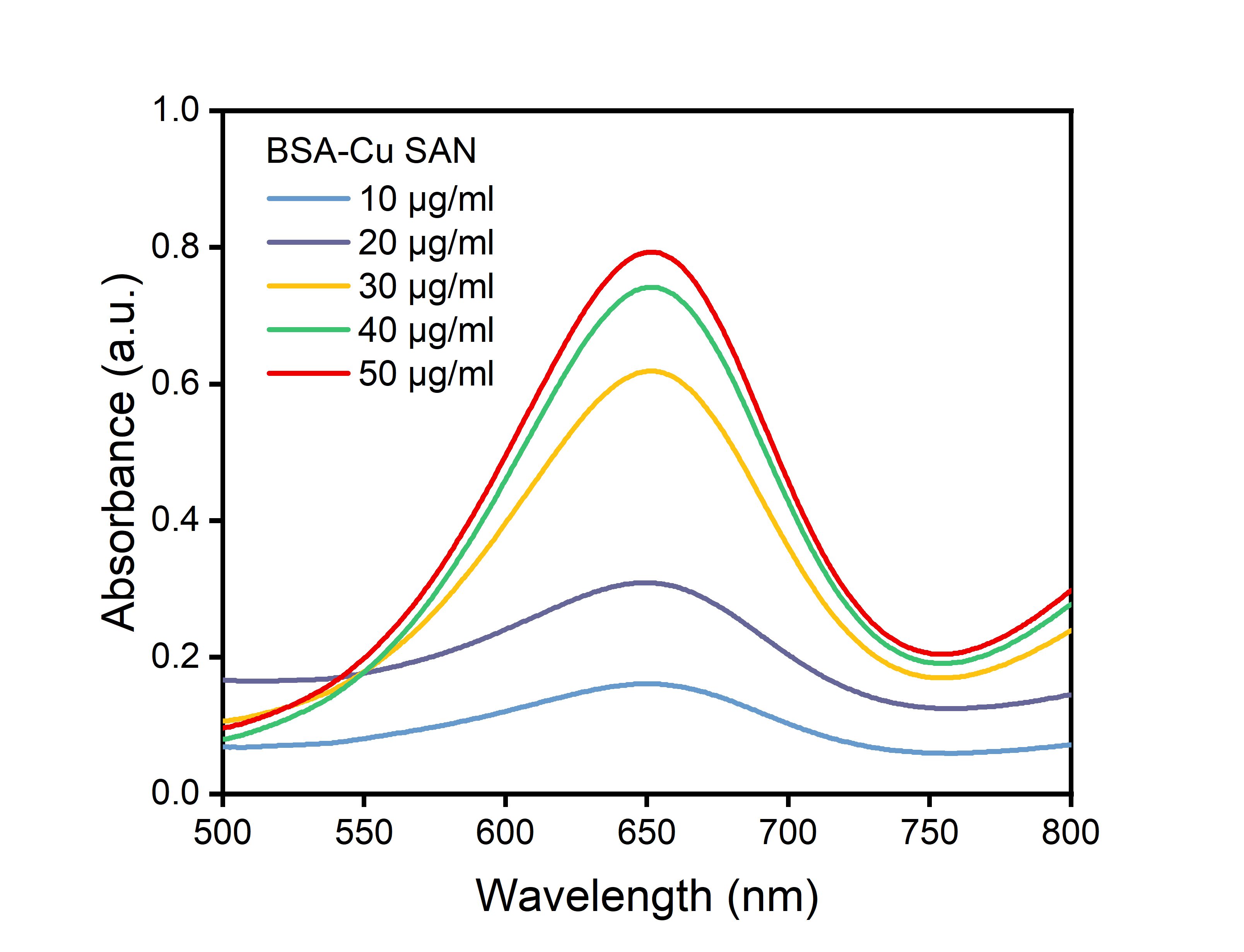


**Figure. S12.** The absorption of TMB in the presence of 1 mM H_2_O_2_ and different concentrations of Cu of BSA-Cu SANs co-incubated for 20min.

Figure. S13.


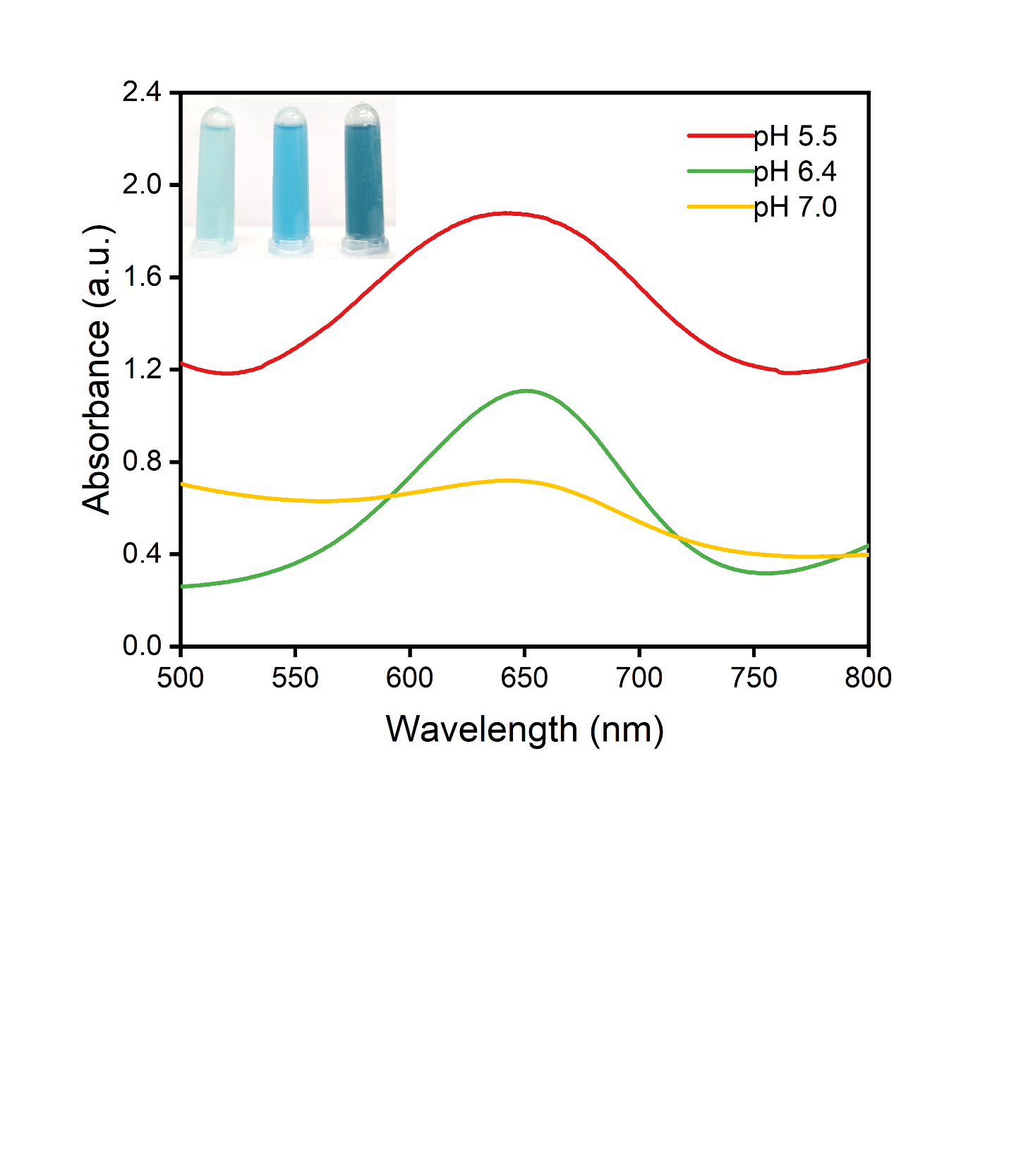


**Figure. S13.** The absorption of TMB in different pH values (7.0, 6.4, and 5.5).

Figure. S14.


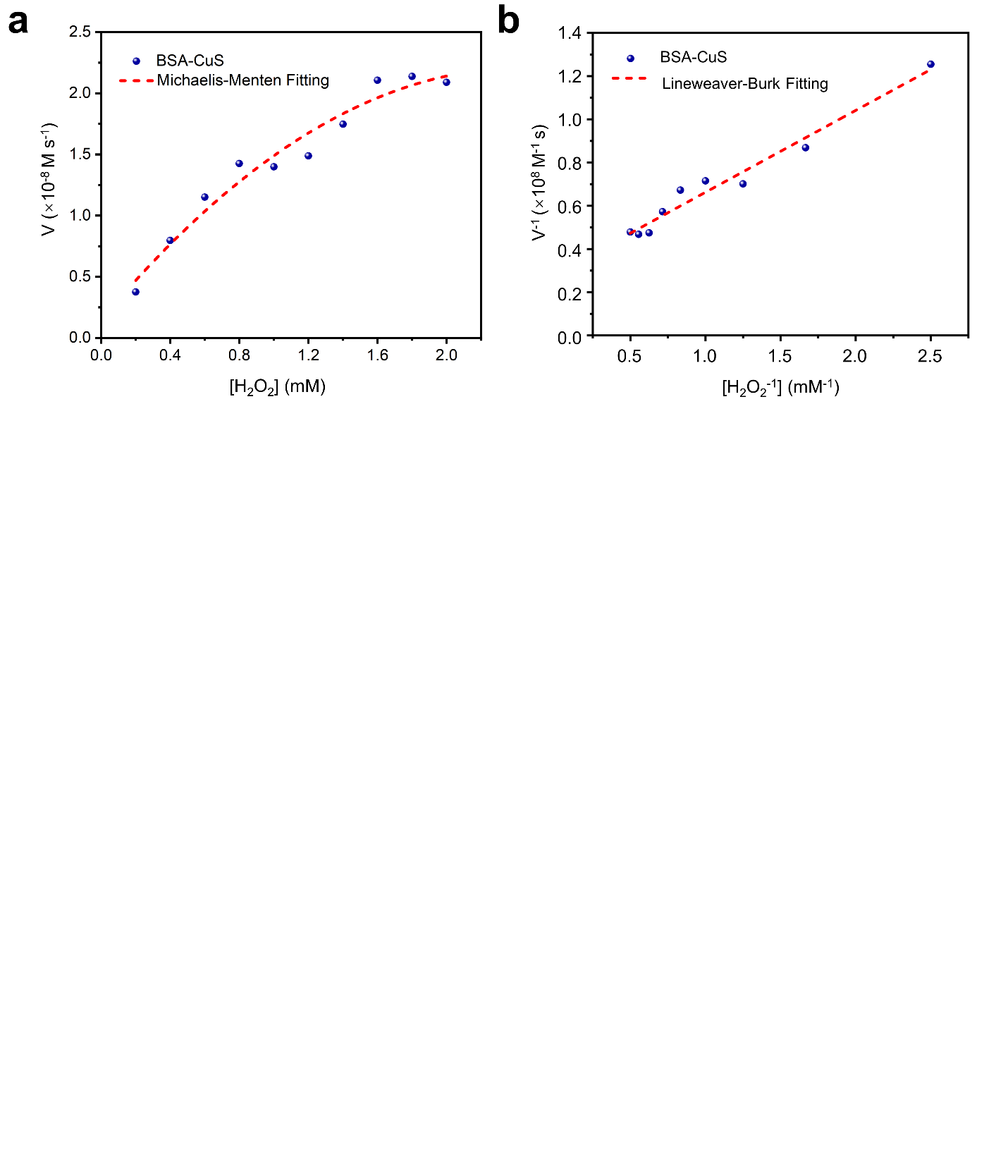


**Figure. S14.** (a) Michaelis-Menten kinetics of BSA-CuS. (b) Lineweaver-Burk plotting of (a).

Figure. S15.


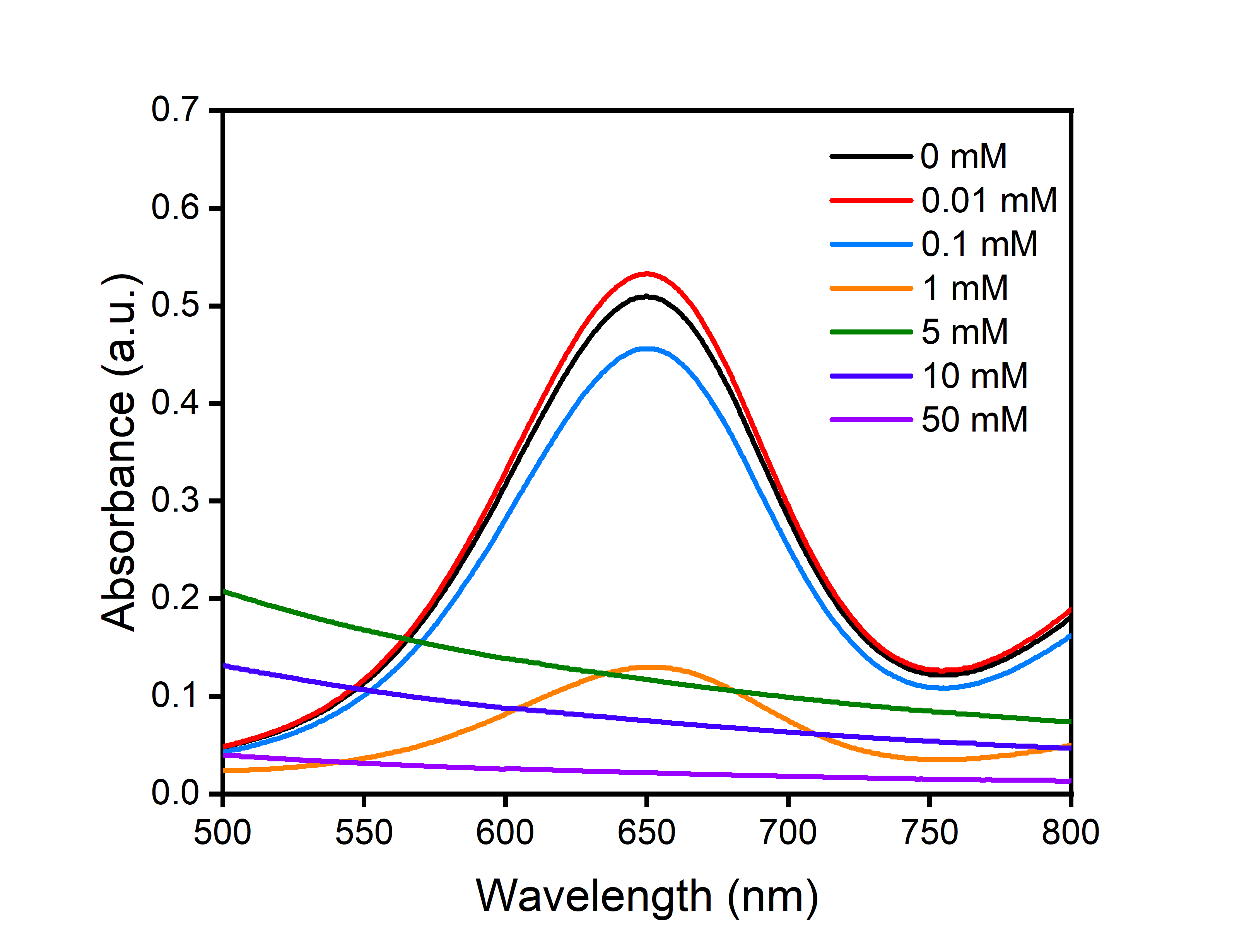


**Figure. S15.** The absorption of TMB reacted with 30 μg/ml Cu of BSA-Cu SAN and 1 mM H_2_O_2_ added in different concentrations of GSH (0-50 mM).

Figure. S16.

**Figure. S16**. The main steps of BSA-Cu SAN catalytic reaction.

Figure. S17.


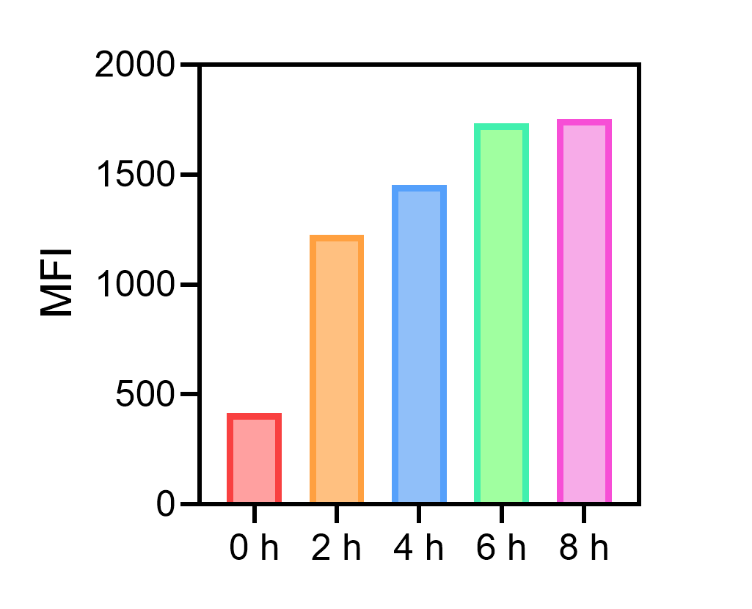


**Figure. S17.** The corresponding mean fluorescence intensity (MFI) values of HCT116 cells uptaking BSA-Cu SAN at different culture times analyzed by flow cytometry.

Figure. S18.


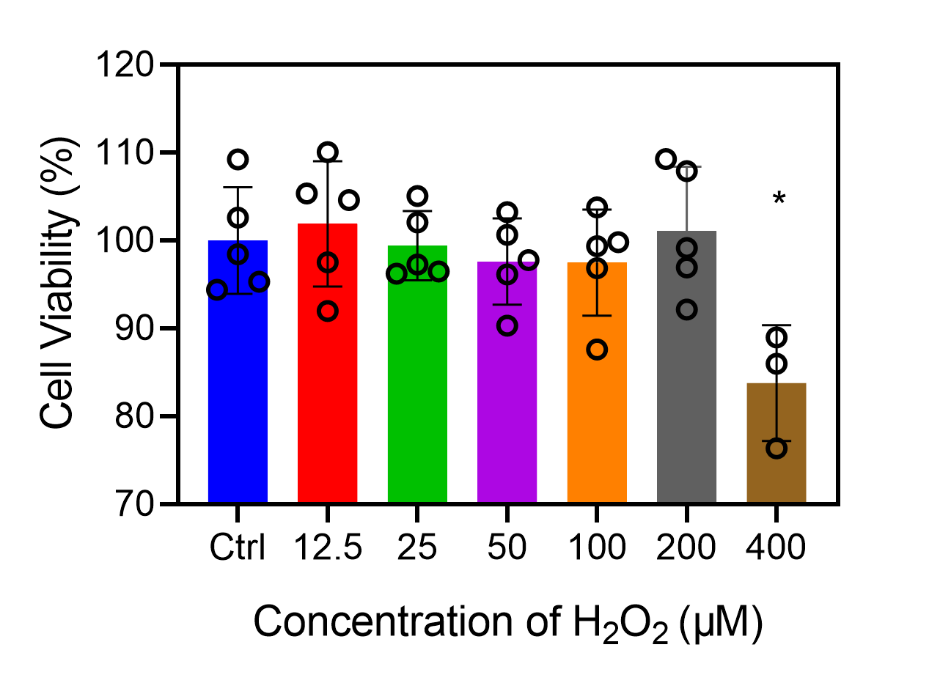


**Figure. S18.** Cell viability of HCT116 cells incubated with different concentrations of H_2_O_2_ for 24 h.

Figure. S19.


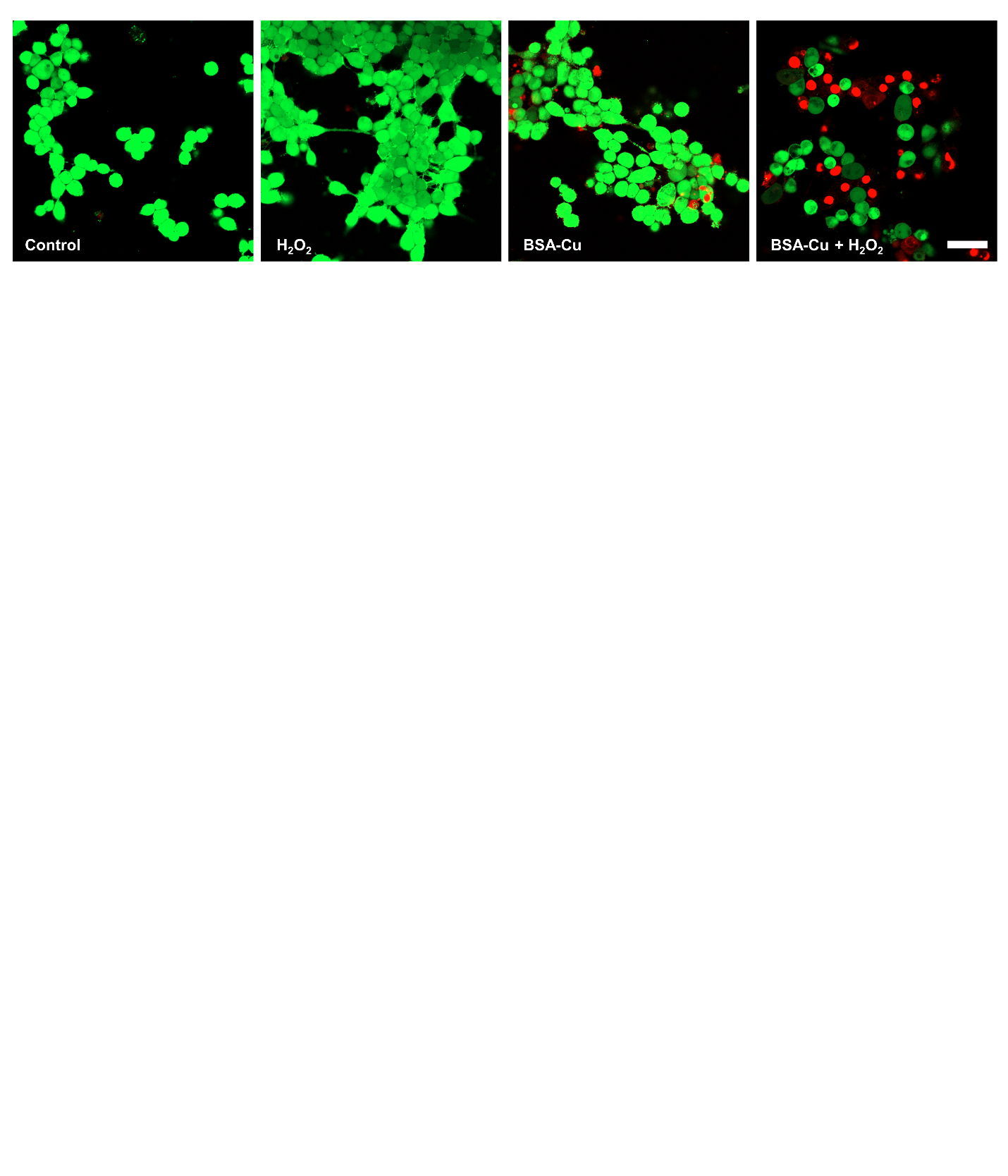


**Figure. S19.** CLSM images of live/dead staining of HCT116 cells treated variously. Calcein-AM-stained green fluorescence and PI-stained red fluorescence represent live and dead cells, respectively. Scale bar = 50 μm.

Figure. S20.


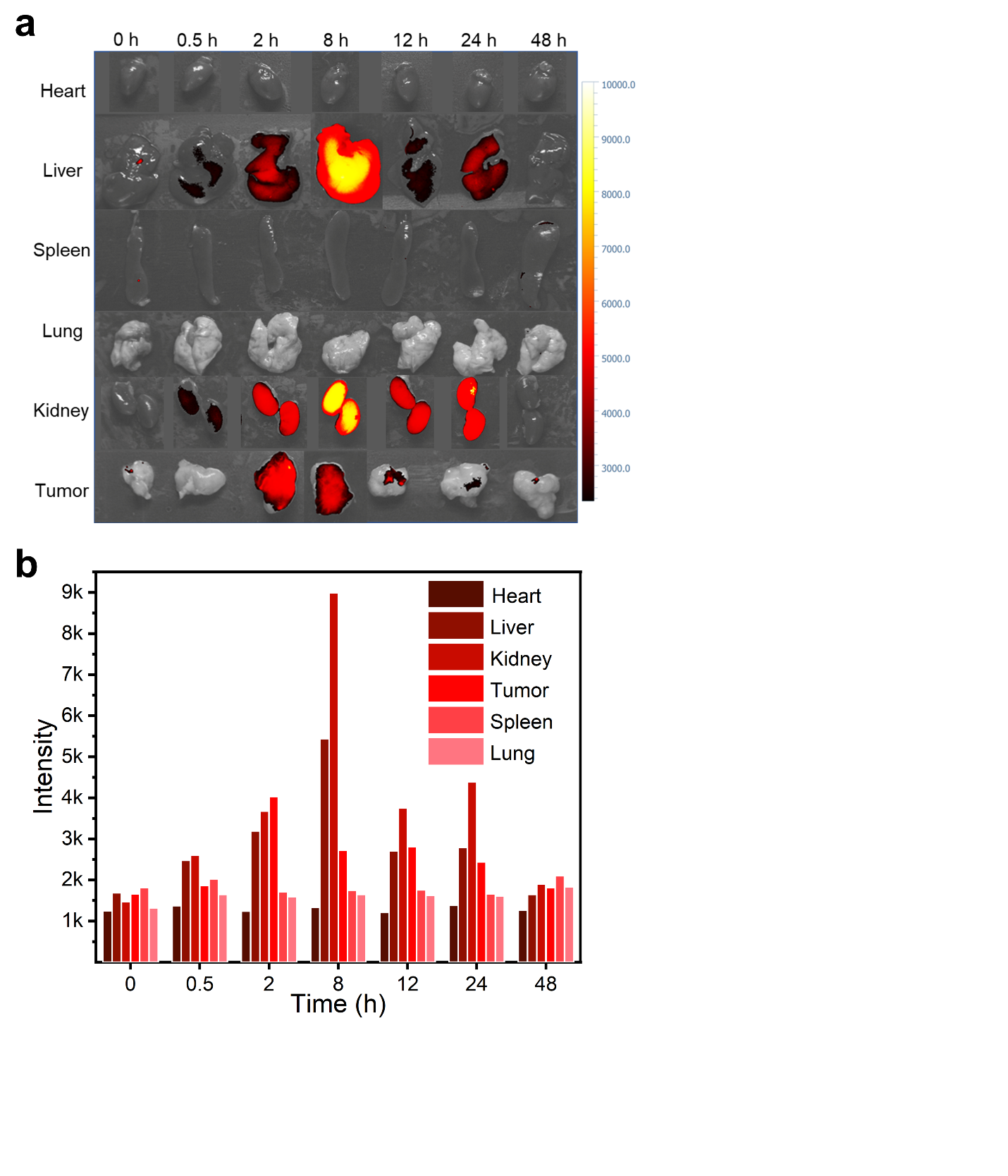


**Figure. S20.** Fluorescent signal of [BSA-Cu-Cy5.5](mailto:BSA@Cu-Cy5.5) in different organs and tumors after intravenously injected into mice. (a) Fluorescent image of organs. (b) Fluorescent intensity of (a).

Figure. S21.


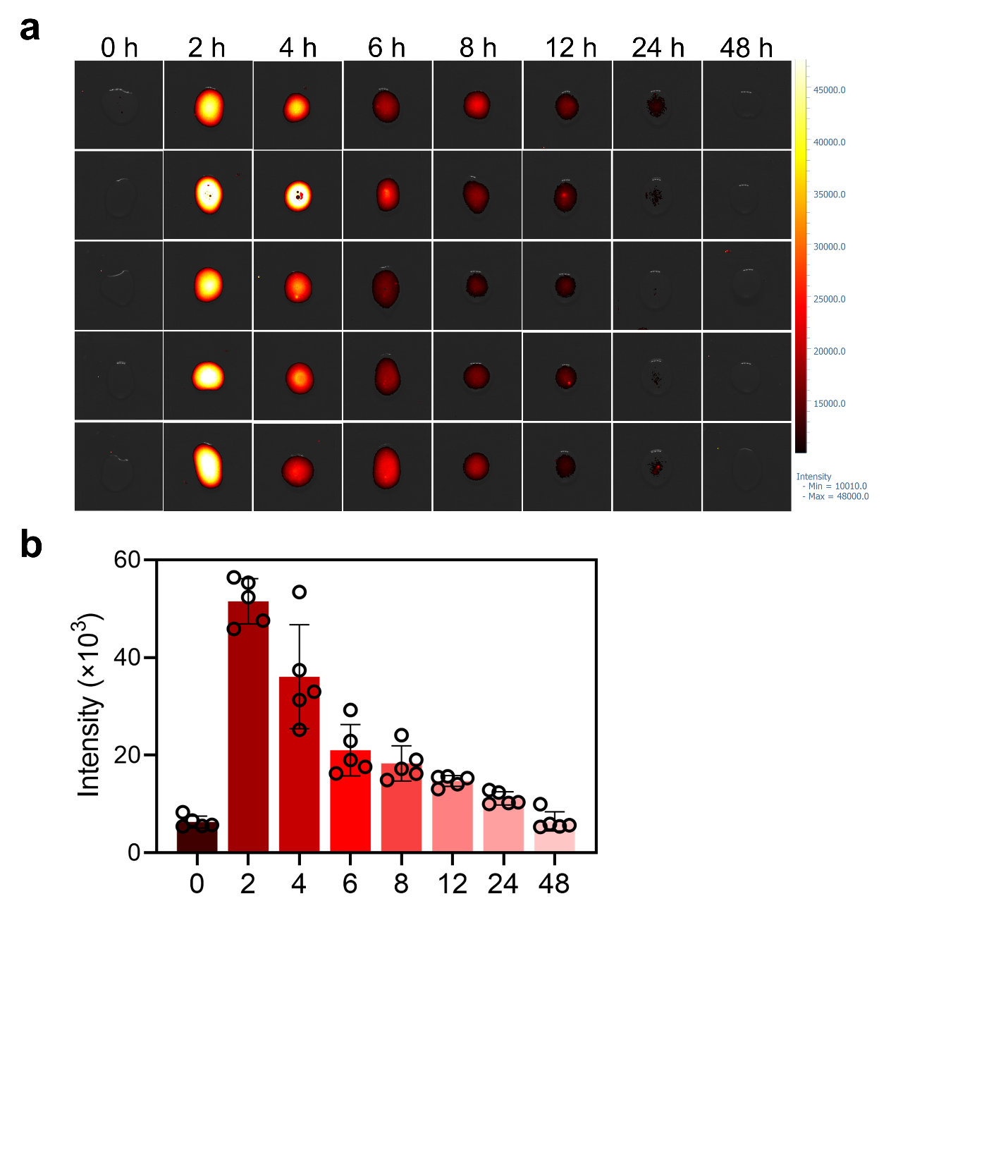


**Figure. S21.** Fluorescent signal of [BSA-Cu-Cy5.5](mailto:BSA@Cu-Cy5.5) in the blood after intravenously injected into mice. (a) Fluorescent image of blood. (b) Fluorescent intensity of (a). n = 5, data represent mean ± SD.

Figure. S22.


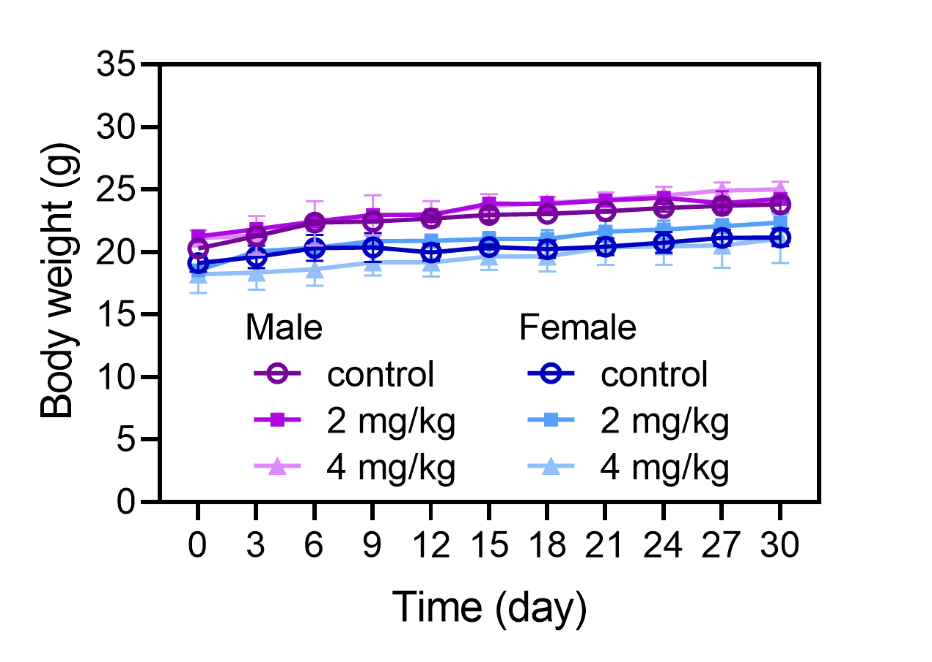


**Figure. S22.** The body weight of BALB/c mice after injected with 100 μl PBS or different concentrations of BSA-Cu SAN intravenously. n = 4, data represent mean ± SD.

Figure. S23.


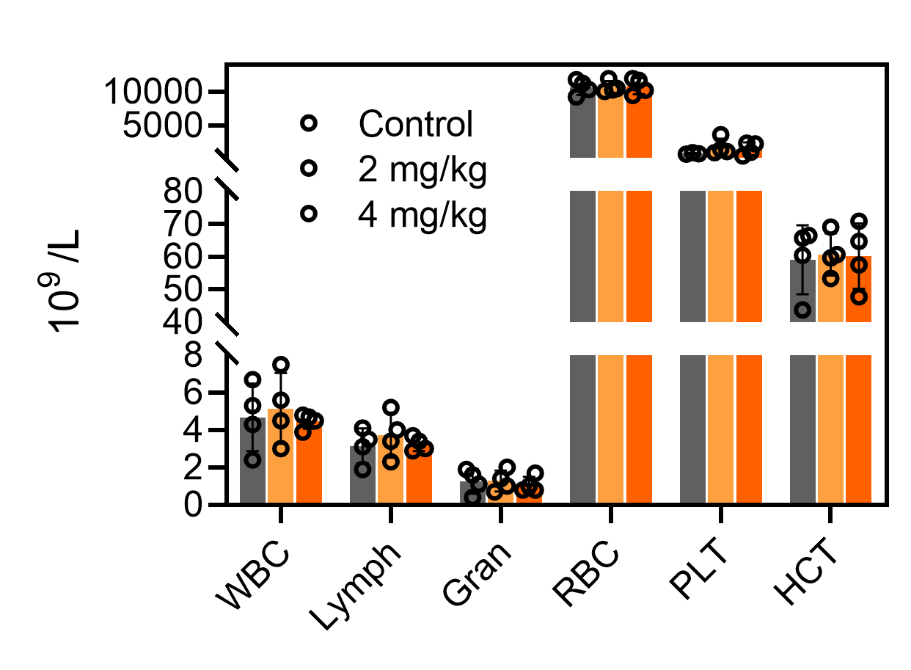


**Figure. S23.** The results of routine blood tests of the BALB/c mice intravenously injected with PBS or different concentrations of BSA-Cu SAN for one month. n = 4, data represent mean ± SD.

Figure. S24.


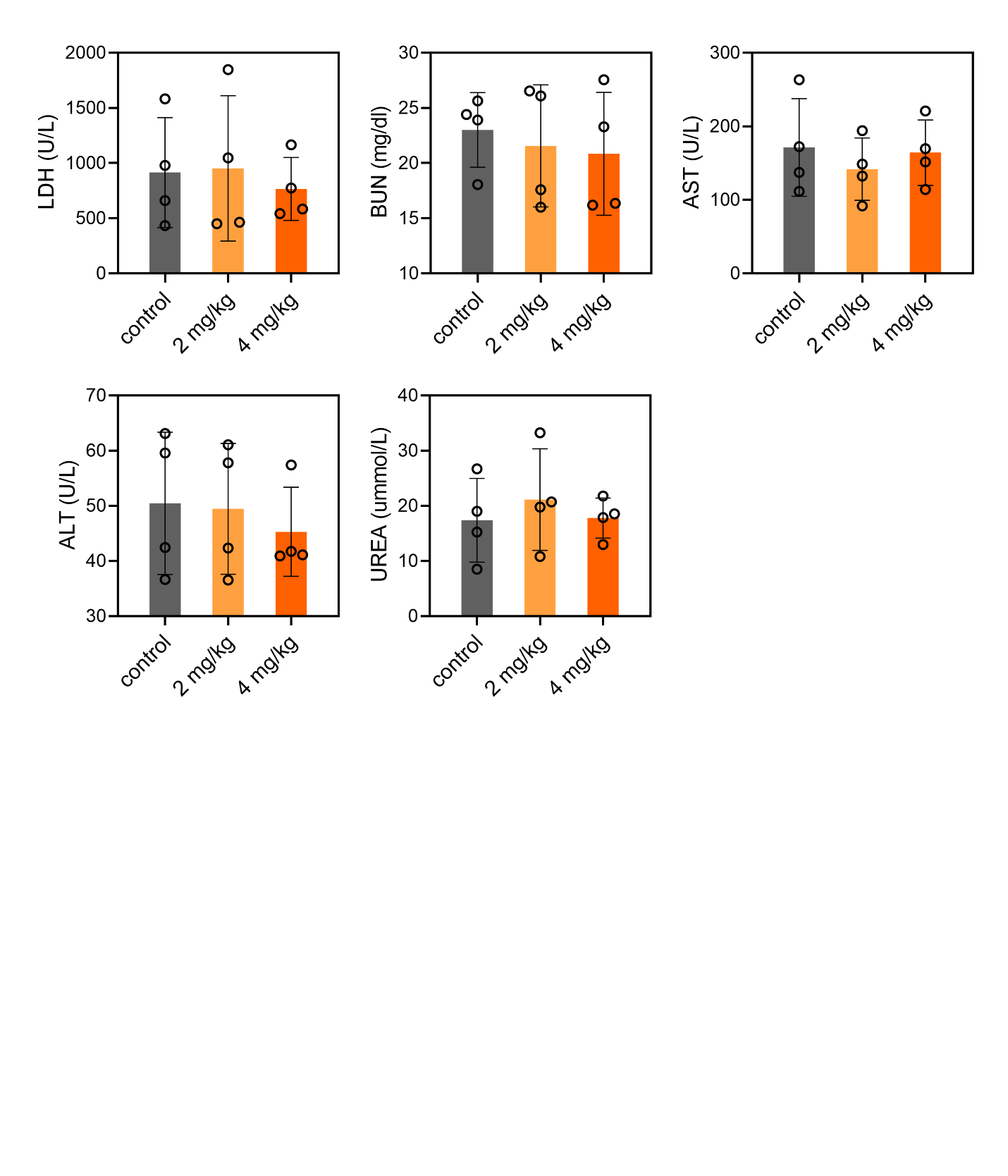


**Figure. S24.** Serum analysis of LDH, BUN, UREA, and ALT of BALB/c mice after intravenously injected with PBS or different concentrations of BSA-Cu SAN for one month. n = 4, data represent mean ± SD.

Figure. S25.


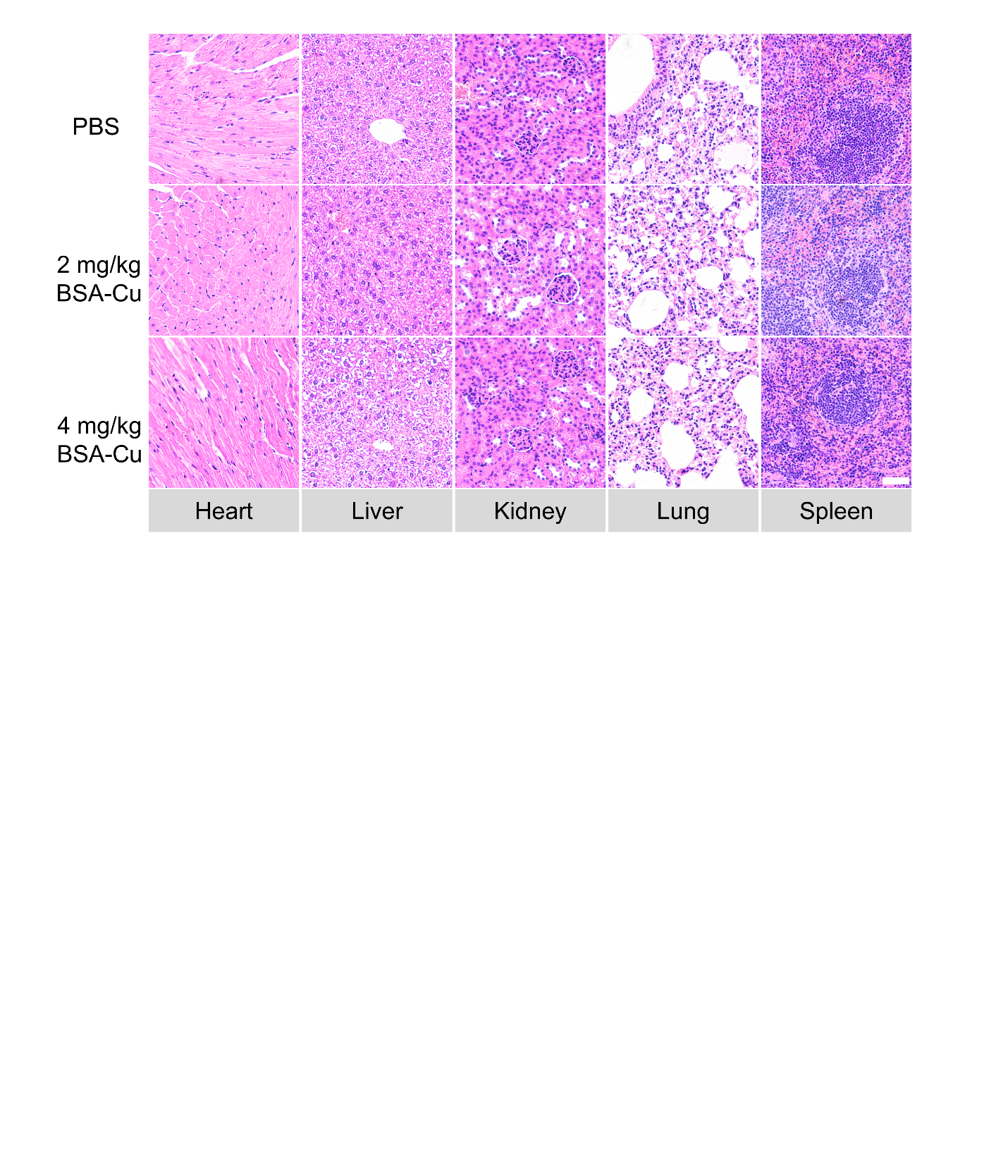


**Figure. S25.** H&E staining of heart, liver, kidney, lung, and spleen of BALB/c mice after treated with PBS or BSA-Cu SAN for one month. Scale bar = 50 μm.

Figure. S26.


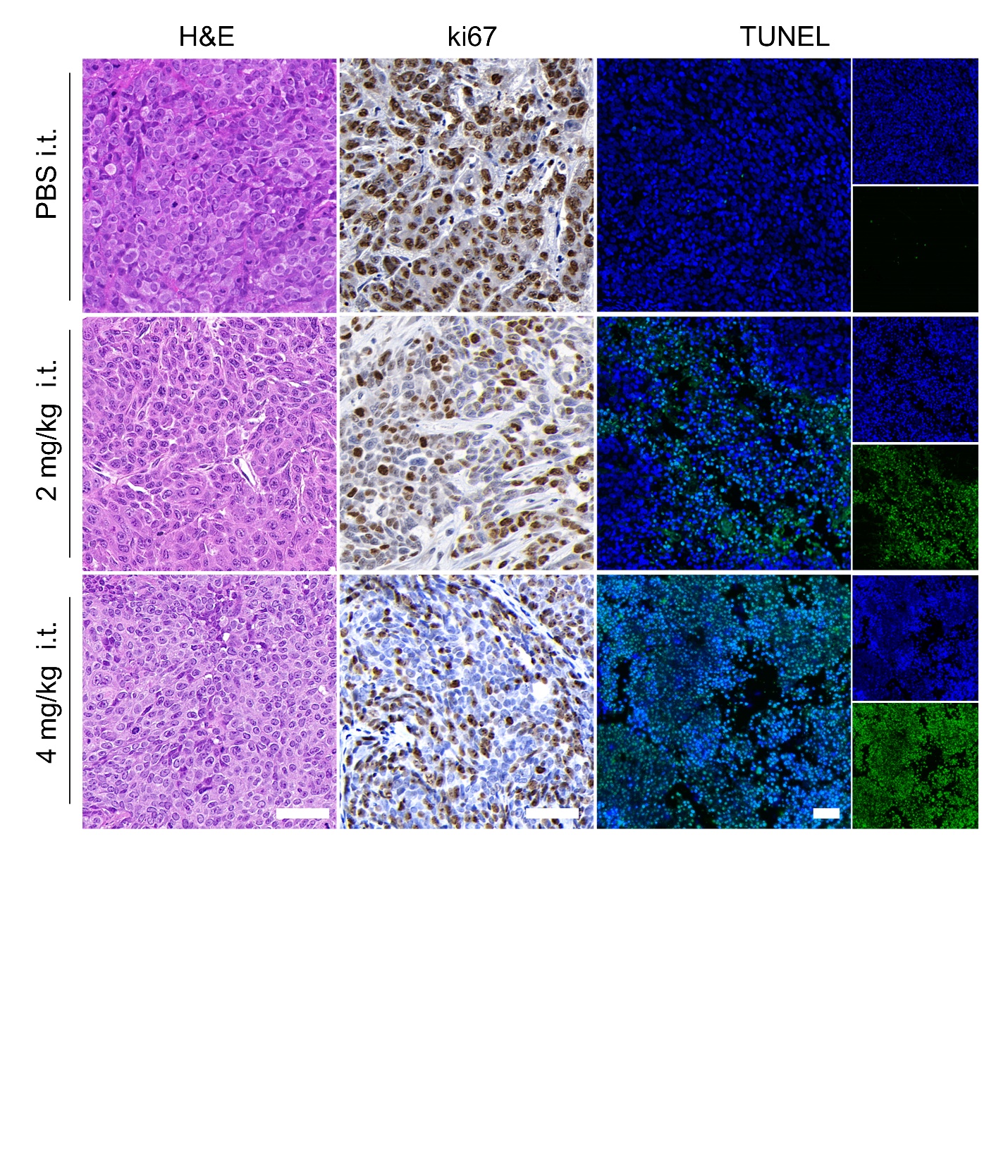


**Figure. S26.** The H&E, ki67, and TUNEL immunostaining of representative tumor tissue sections intratumorally injected with PBS or different concentrations of BSA-Cu SAN. Scale bar = 50 μm.

Figure. S27.


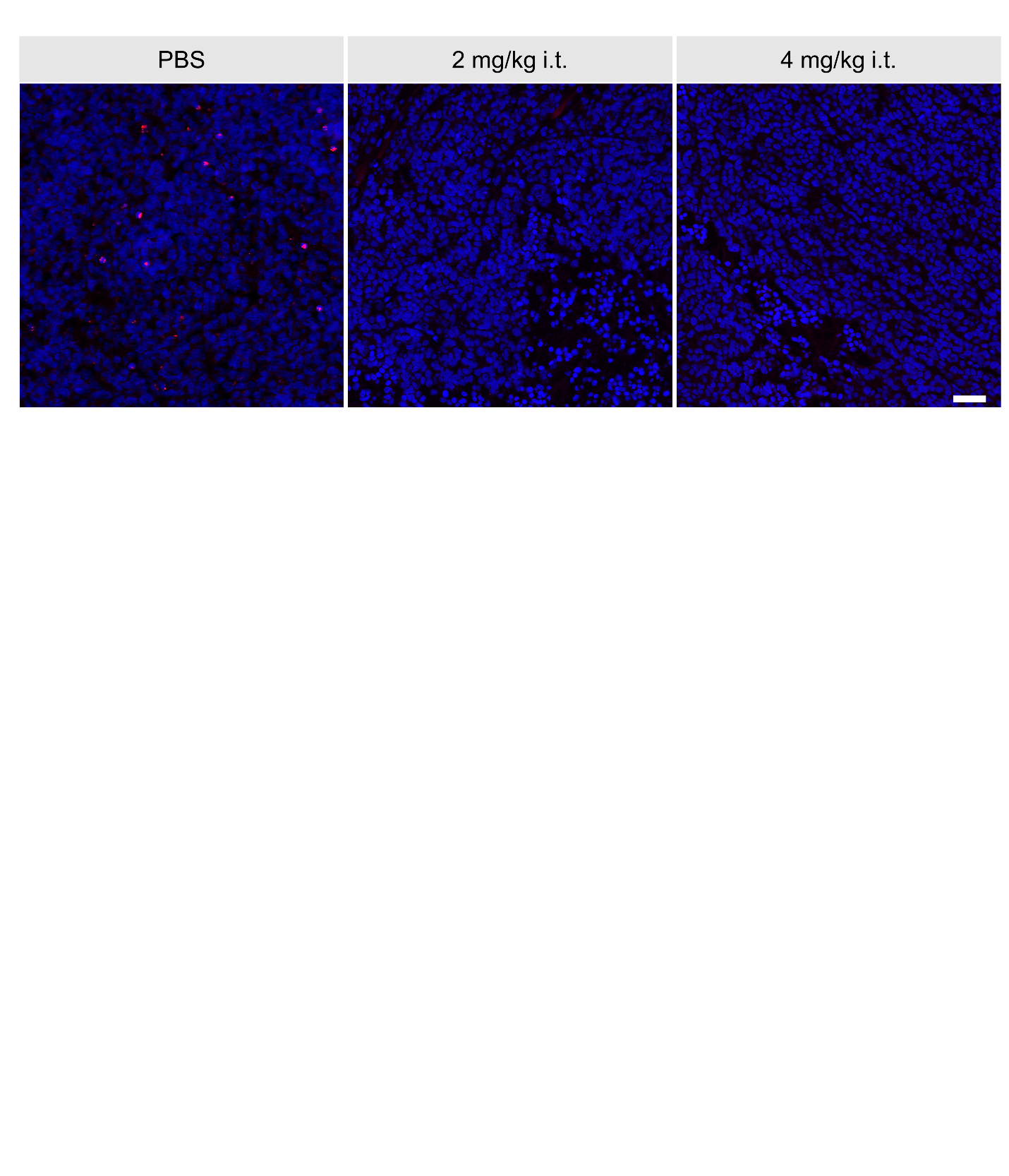


**Figure. S27.** The images of tumor tissue section in different groups in which the nucleic acid stained blue with DAPI and red for *F. nucleatum* probe. Scale bar = 50 μm.

Figure. S28.


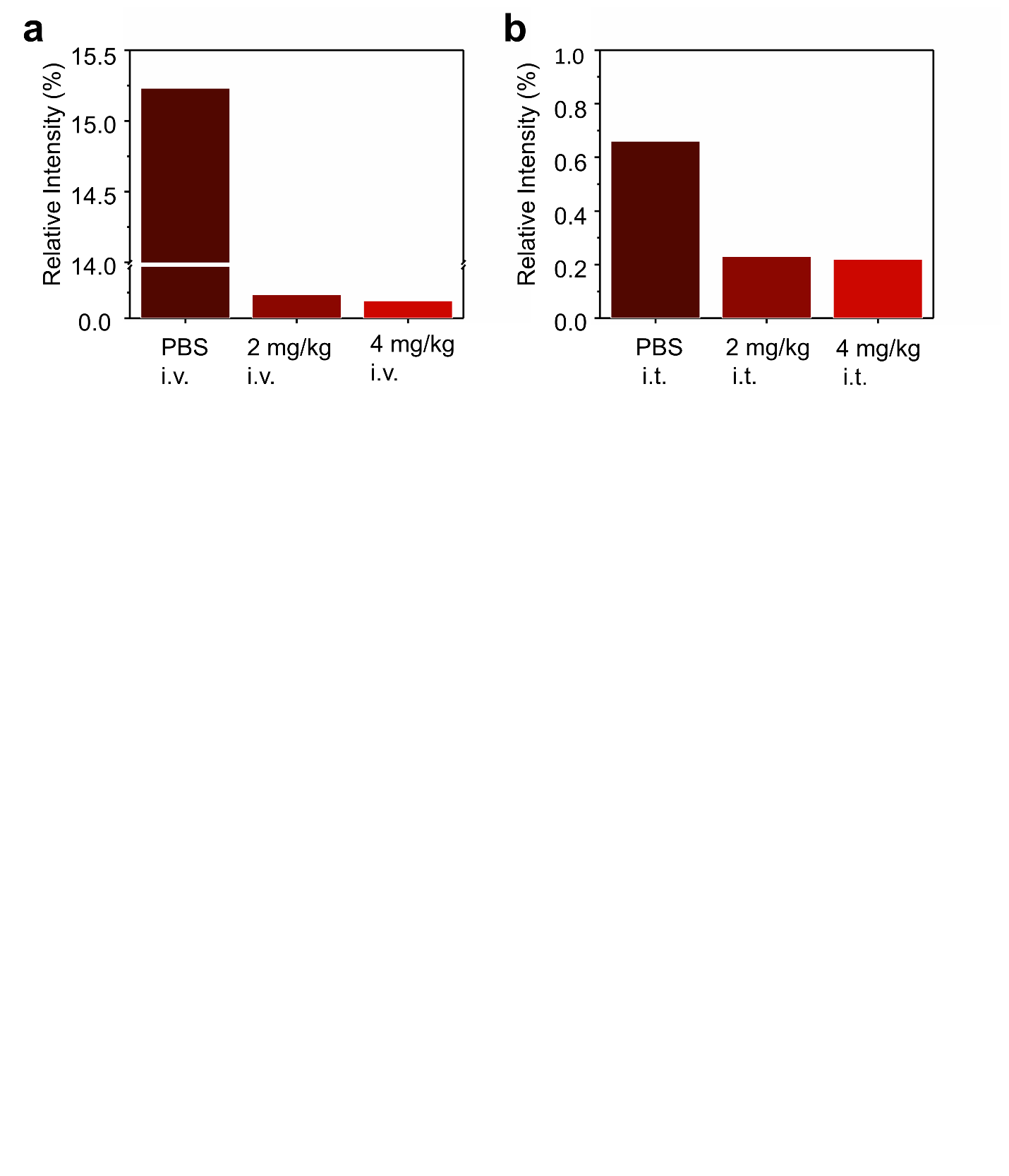


**Figure. S28.** Semi-quantitative analysis of intratumoral *F. nucleatum* in (a) Fig. 6i and (b) Supplementary Fig. 27.

Table S1.

The prediction of Cu^+^ binding site of BSA. The results are rank-ordered according to their docking scores

| Rank | Binding site | Score |
| --- | --- | --- |
| 1 | 67H, 246H, 248D, H_2_O | -5.286 |
| 2 | 6E, 9H | -5.025 |
| 3 | 287H, 291E | -4.006 |
| 4 | 67H, 246H | -3.601 |
| 5 | 195Q, 199C, 241H, 244C | -3.425 |
| 6 | 9H, 251E, 254D | -3.403 |
| 7 | 243E, 246H | -3.353 |
| 8 | 3H, 6E, 9H | -3.348 |
| 9 | 147Y, 149Y, 246H | -3.345 |
| 10 | 9H, 13D | -3.146 |

Table S2.

EXAFS fitting parameters at the Cu K-edge for various samples (*Ѕ*_0_^2^=0.904)

| Sample | Shell | *CN^a^* | *R*(Å)*^b^* | *σ*^2^(Å^2^)*^c^* | Δ*E*_0_(eV)*^d^* | *R* factor |
| --- | --- | --- | --- | --- | --- | --- |
| *Cu K-edge* | | | | | | |
| Cu foil | Cu-Cu | 12* | 2.54±0.01 | 0.0085±0.0004 | 4.7±0.6 | 0.0041 |
| Cu_2_O | Cu-O | 1.6±0.1 | 1.85±0.01 | 0.0023±0.0008 | 9.1±1.1 | 0.0086 |
|  | Cu-Cu | 12.0±0.4 | 3.05±0.01 | 0.0218±0.0008 |  |  |
| sample | Cu-N/O | 4.2±0.5 | 1.95±0.01 | 0.0068±0.0024 | 6.8±1.5 | 0.0139 |
|  | Cu-N | 2.2±0.2 | 1.89±0.01 | 0.0027±0.0018 | 3.7±3.9 | 0.0084 |
|  | Cu-O | 1.7±0.4 | 2.02±0.02 |  | 2.8±3.2 |  |

Table S3.

Comparison of the catalytic performance based on the contents of Cu active sites in the BSA-Cu SAN and other copper containing nanozymes, including BSA-CuS, Cu_x_O as well as Cu SANs. HRP is also listed as a commonly used reference.

| Nanozymes | [E/Cu]  (mM) | *K_m_*  (mM) | *V_max_*  (mMs^-1^) | *k_cat_*  (s^-1^) | *k_cat_/K_m_*  (mM^-1^s^-1^) | Refs. |
| --- | --- | --- | --- | --- | --- | --- |
| BSA-Cu | 4.69×10^-1^ | 0.48 | 1.71×10^-4^ | 3.65×10^-3^ | 7.60×10^-4^ | This work |
| BSA-CuS | 4.69×10^-1^ | 1.33 | 3.52×10^-5^ | 7.10×10^-5^ | 5.34×10^-5^ | This work |
| Glu-Cu-CPDs | 7.81 | 1.81 | 6.97×10^-5^ | 8.92×10^-6^ | 4.93×10^-6^ | *Chem. Int. Ed*. 2023, 62, e202214042 |
| Cu SAs/CN | 1.20×10^-6^ | 0.45 | 1.77×10^-4^ | 1.48×10^2^ | 3.28×10^2^ | *Adv. Sci.* 2022, 9,  2103977 |
| Cu-N-C | 2.68×10^-3^ | 19.94 | 2.01×10^-4^ | 7.50×10^-2^ | 3.80×10^-3^ | *Anal. Chem.* 2020, 92, 4, 3373-3379 |
| MoO_x_-Cu-Cys | 8.18×10^-2^ | 2.02 | 1.71×10^1^ | 2.09×10^2^ | 1.03×10^2^ | *J. Am. Chem. Soc.* 2023, 145, 7, 4279-4293 |
| Cu_x_O | 5.24×10^-2^ | 52.3 | 2.37×10^-4^ | 4.52×10^-3^ | 8.65×10^-5^ | *J. Am. Chem. Soc.* 2019, 141, 2, 1091-1099 |
| HRP | 1.00×10^-6^ | 5.55 | 1.06×10^-3^ | 1.06×10^3^ | 1.91×10^2^ | *Nat Catal* 2021,4(5),  407-417 |

Table S4.

Calculated absolute energies and energy corrections for the stationary points of the wild type

|  | E_BS1_  (a.u.) | E_BS2_  (a.u.) | E_solvation_  (a.u.) | E_ZPE_  (a.u.) | ΔE_total_  (kcal/mol) |
| --- | --- | --- | --- | --- | --- |
| RC | -109.934196 | -110.234756 | -0.0206162 | 0.303258 | 0.0 |
| Int1 | -261.514388 | -261.872457 | -0.0229226 | 0.330723 | -13.4 |
| TS1 | -261.480367 | -261.840321 | -0.0236996 | 0.330723 | 4.4 |
| Int2 | -185.761491 | -186.090715 | -0.0248389 | 0.318698 | -9.2 |
| Int3 | -186.205652 | -1186.5223 | -0.0569108 | 0.331968 | -21.6 |
| Int4 | -109.749367 | -110.035662 | -0.0074251 | 0.306061 | -13.4 |
